# Dopamine Reduces SARS-CoV-2 Replication In Vitro through Downregulation of D2 Receptors and Upregulation of Type-I Interferons

**DOI:** 10.3390/cells11101691

**Published:** 2022-05-19

**Authors:** Fiona Limanaqi, Silvia Zecchini, Borana Dino, Sergio Strizzi, Gioia Cappelletti, Olga Utyro, Claudia Vanetti, Micaela Garziano, Irma Saulle, Mario Clerici, Mara Biasin

**Affiliations:** 1 Department of Pathophysiology and Transplantation, University of Milan, Via Francesco Sforza, 20122 Milan, Italy; micaela.garziano@unimi.it (M.G.); mario.clerici@unimi.it (M.C.); 2 Department of Biomedical and Clinical Sciences L. Sacco, University of Milan, Via G.B. Grassi, 20122 Milan, Italy; silvia.zecchini@unimi.it (S.Z.); borana.dino@studenti.unimi.it (B.D.); sergio.strizzi@unimi.it (S.S.); gioia.cappelletti@unimi.it (G.C.); olga.utyro@unimi.it (O.U.); claudia.vanetti@unimi.it (C.V.); irma.saulle@unimi.it (I.S.); 3 IRCCS Fondazione Don Carlo Gnocchi, 20148 Milan, Italy

**Keywords:** dopamine, D2DR, interferons, quinpirole, NLRP3 inflammasome

## Abstract

Recent evidence suggests that SARS-CoV-2 hinders immune responses via dopamine (DA)-related mechanisms. Nonetheless, studies addressing the specific role of DA in the frame of SARS-CoV-2 infection are still missing. In the present study, we investigate the role of DA in SARS-CoV-2 replication along with potential links with innate immune pathways in CaLu-3 human epithelial lung cells. We document here for the first time that, besides DA synthetic pathways, SARS-CoV-2 alters the expression of D1 and D2 DA receptors (D1DR, D2DR), while DA administration reduces viral replication. Such an effect occurs at non-toxic, micromolar-range DA doses, which are known to induce receptor desensitization and downregulation. Indeed, the antiviral effects of DA were associated with a robust downregulation of D2DRs both at mRNA and protein levels, while the amount of D1DRs was not significantly affected. While halting SARS-CoV-2 replication, DA, similar to the D2DR agonist quinpirole, upregulates the expression of ISGs and Type-I IFNs, which goes along with the downregulation of various pro-inflammatory mediators. In turn, administration of Type-I IFNs, while dramatically reducing SARS-CoV-2 replication, converges in downregulating D2DRs expression. Besides configuring the CaLu-3 cell line as a suitable model to study SARS-CoV-2-induced alterations at the level of the DA system in the periphery, our findings disclose a previously unappreciated correlation between DA pathways and Type-I IFN response, which may be disrupted by SARS-CoV-2 for host cell invasion and replication.

## 1. Introduction

Since the outbreak of Coronavirus Disease 19 (COVID-19) by the novel Severe Acute Respiratory Coronavirus-2 (SARS-CoV-2), immense efforts have been made by the scientific community to uncover its viral mechanisms of action, potential therapeutic targets, and factors controlling the susceptibility/outcome of the infection. A decrease in the innate antiviral response, hyper-inflammation, along with exhaustion/loss in function of T-cells are considered major determinants of COVID-19 severity.

Recent intriguing evidence suggests that SARS-CoV-2 may hinder immune responses via dopamine (DA)-related mechanisms [1,2,3]. On the one hand, dopamine (DA) receptors (DRs) have been hypothesized to be exploited by the virus to improve both its entry and life cycle within cells; on the other, SARS-CoV-2 was documented to downregulate L-Dopa-Decarboxylase (DDC), the rate-limiting enzyme for the conversion of L-DOPA to DA [3]. This is key in the frame of CNS viral infections and pre-existing comorbidities, as well as virus-related neurological manifestations, which commonly appear after the diagnosis of COVID-19 [4]. However, besides mere neurological manifestations, DA-related alterations may lead to a plethora of systemic effects which may be relevant in the frame of COVID-19 pathogenesis [5,6,7]. Indeed, DA or DA agonists, apart from modulating reward, motivation and movement, exert endocrine, respiratory, cardiovascular, renal, gastrointestinal, and immune functions by acting on G protein-coupled D1-like (D1, D5) and D2-like (D2, D3, D4) receptors, which are abundantly expressed in a variety of immune cell subpopulations and organs [5,6,8,9,10,11,12,13]. These include alveolar epithelial lung cells, the major site of SARS-CoV-2 infection [12,13]. As documented by pioneer studies, the lungs are endowed with scattered neuroendocrine and neuroepithelial bodies, that are maximally developed around birth [13]. Coupled with evidence about the presence of both DRs and DDC within these cells, this configures the respiratory epithelium as an important potential source of extra-neuronal DA besides chromaffin cells of the adrenal medulla and the gastrointestinal tract [12,13]. Thus, it is not surprising that alterations in DA synthetic pathways or abnormal stimulation/expression of D1- and D2-like receptors may disrupt a variety of peripheral physiological functions. These include also innate and adaptive immunity, leading to exacerbation of pro-inflammatory responses and worsening of various pathological scenarios.

Supporting a role for DA pathways in SARS-CoV-2 infection, a variety of indirect and direct DA modulators, including the DA precursor L-DOPA, have been ranked among the top drugs that own the greatest potential to interact with SARS-CoV-2 replication [14,15,16,17,18]. Nonetheless, studies addressing the specific role of DA in the frame of SARS-CoV-2 infection are still missing.

Based on these premises, in the present study, we investigate the role of DA in SARS-CoV-2 infection along with potential links with innate immune pathways in CaLu-3 human lung epithelial cells, which are widely used as a model for SARS-CoV-2 infection in the light of their high viral susceptibility and permissibility to effective replication. Remarkably, within these cells, we could detect the expression of both DA synthetic enzymes and DRs, which makes them a suitable model to study SARS-CoV-2-induced alterations at the level of the DA system. We document here for the first time that, DA in the non-toxic micromolar range (10–100 μM), dose-dependently reduces viral replication through downregulation of D2 DA receptors (D2DRs), likely by acting at both transcriptional, and post-translational levels. In this frame, we also disclose a role for Type-I IFNs (α, β) bridging D2DR downregulation and inhibition of SARS-CoV-2 replication.

Our findings are expected to pave the way for future research centered on the role of the DA system as a potential therapeutic target against COVID-19 multi-organ pathology, which deserves to be investigated in other cell types and animal models.

## 2. Materials and Methods

### 2.1. Cell Lines, Virus, and Reagents

CaLu-3 cells (HTB-55™, human epithelial cells from lung adenocarcinoma) and VeroE6 (CRL-1586™, African green monkey kidney epithelial cells) were purchased from American Type Culture Collection (ATCC^®^, Manassas, VA, USA). A549 (NR-53522, human lung adenocarcinoma cells) expressing Human Angiotensin-Converting Enzyme 2 (A549-hACE2) were obtained through BEI Resources, NIAID, NIH. CaLu-3 cells were grown in DMEM high glucose (ECB20722L, Euroclone, Milan, Italy) supplemented with 10% FBS, 1% L-Glutamine and PenStrep, and 1% NEAA. VeroE6 cells and A549-hACE2 cells were grown in DMEM high glucose (ECB20722L, Euroclone, Milan, Italy), supplemented with 10% FBS, and 1% L-Glutamine and PenStrep. Cells were grown at 37 °C in 5% CO_2_ and at 98% humidity. Cells were routinely checked for mycoplasma contamination by PCR test. Cells between passages 15 and 25 were used for the experiments.

The following reagents were used to stimulate cell culture: Dopamine Hydrochloride (Merck-Sigma, Milan, Italy); D1DR and D2DR agonists or antagonists (D1DR agonist SKF38393 Hydrochloride; D1DR antagonist SCH 23,390 Hydrochloride; D2DR agonist Quinpirole Hydrochloride, D2DR antagonist Eticlopride Hydrochloride, Merck-Sigma, Milan, Italy); Type-I Interferons (Human Interferon Beta NR-3080 and Human Recombinant Alpha A NR-3083, obtained from BEI Resources, NIAID, NIH). To prepare stock solutions, all the compounds except for DA were dissolved in sterile PBS w/o calcium and magnesium, aliquoted, and stored at −20 °C (IFN-α, 12.000 IU/mL, IFN-β 15.000 IU/mL, D1DR agonist 10 mM, D1DR antagonist 100 mM, D2DR agonist 28 mM, D2DR antagonist 40 mM). On the day of the experiment, stock solutions were further diluted in the same vehicle to obtain the final concentrations for cell culture treatment, as specified in Section 2.2. DA stock solution was freshly prepared the day of the experiment by dissolving 40 mg of DA powder in 10 mL of sterile PBS *w*/*o* calcium and magnesium (pH adjusted to 6.5 to prevent readily DA oxidation, at a final concentration of 21 mM), and appropriate dilutions were performed in the same vehicle to obtain the concentrations for cell culture treatment, as specified in Section 2.2. As per manufacturer instruction, light exposure was avoided during the preparation of DA and DR agonist/antagonist solutions and subsequent experiments with these compounds.

### 2.2. In Vitro SARS-CoV-2 Infection Assay and Treatment Protocols

For infection assays, SARS-CoV-2 Virus Human 2019-nCoV (strain 2019-nCoV/Italy-INMI1, Rome, Italy) was expanded in VeroE6 cells and infectious viral particle concentration was assessed by TCID_50_ endpoint dilution assay in CaLu-3 as previously described. [19]. In detail, CaLu-3 cells were plated onto 96-well plates (3 × 10^4^ cells/well) for 48 h and incubated with SARS-CoV-2 serial 10-fold dilutions, from 10^6^ to 10^−4^ TCDI_50_/mL (50 μL) for 3 h at 37 °C in 5% CO_2_. Cells were washed in PBS to remove unbound virus and incubated at 37 °C in 5% CO_2_ for 72 h. Viral titer from cell supernatants was determined to assess TCID_50_ through a single-step, real-time, quantitative Reverse Transcriptase-Polymerase Chain Reaction (RT-qPCR, detailed in Section 2.2). All the experiments with the SARS-CoV-2 virus were performed in the BSL3 facility; before sample analysis outside the BSL3 area, the virus was inactivated according to institutional safety guidelines.

CaLu-3 cells were seeded and cultured for 48 h in either 12-well plates (2.5 × 10^5^ cells/well in a final volume of 1 mL for assessment of viral replication, Trypan Blue exclusion assay, and intracellular RNA extraction), 24-well plates (1.25 × 10^5^ cells/well in a final volume of 500 μL for assessment of viral replication and immunofluorescence experiments), or 96-well plates (3 × 10^4^ cells/well in a final volume of 100 mL for MTT cell viability assay both in the absence and presence of SARS-CoV-2). A549-hACE2 cells were seeded and cultured for 24 h in 12-well plates (2 × 10^5^ cells for assessment of viral replication and cellular RNA extraction).

Cells were treated according to a sub-chronic administration protocol. In detail, prior to either Mock (Uninfected) or SARS-CoV-2 infection, cells were treated with DA (from 10 nM to 300 μM, for 1 h), D1DR, and D2DR agonists or antagonists (10 or 50 μM, either alone or 30 min prior to administration of DA), or Type-I Interferons (10 or 100 IU/mL, for 16 h). Control cells were treated with an equal volume of vehicle (PBS, 20 μL in a final volume of 1 mL for 12-well plates, 10 μL in a final volume of 500 μL for 24-well plates, and 2.5 μL in a final volume of 100 μL for 96-well plates). At these time points, the medium was removed, and the cells were either Mock-infected (in DMEM supplemented with 2% FBS in the absence of the virus) or infected with SARS-CoV-2 (MOI 0.02, virus suspended in DMEM supplemented with 2% FBS) for 3 h. All the compounds (DA, D1/D2DR agonists/antagonists, or Type-I IFNs) were added during the infection at the above-indicated concentrations. After the infection, cells were washed in PBS to remove unbound virus and replenished with DMEM 10% FBS supplemented with the specific treatments (DA, D1/D2DR agonists/antagonists, or Type-I IFNs) at the corresponding concentrations. Control cells (both Uninfected (Mock)- and SARS-CoV-2-infected) were treated with an equal volume of PBS (both during infection and post-infection). All the supernatants and cells were harvested at 24 and/or 48 h post-infection and appropriately stored for further processing as specified below.

### 2.3. RNA Extraction, Reverse Transcription, and Gene Expression

#### 2.3.1. Viral Replication

For assessment of viral replication at 24 and 48 h post-infection, RNA from cell culture supernatants was extracted by using Maxwell RSC Viral Total Nucleic Acid Purification Kit (Promega, Fitchburg, WI, USA) through the Maxwell^®^ RSC Instrument (Promega, Fitchburg, WI, USA). Viral RNA was quantified as previously described [20]. Briefly, single-step, real-time, RT-qPCR (GoTaq^®^ 1-Step RT-qPCR, Promega, Fitchburg, WI, USA) and the 2019-nCoV CDC qPCR Probe Assay kit with probes targeting SARS-CoV-2 nucleocapsid (N) gene and the human RNase P gene as an internal control (IDT, Coralville, IA, USA) were used on a CFX96 instrument (Bio-Rad, Hercules, CA, USA). Absolute viral copy number quantification was performed by generating a standard curve from the quantified 2019-nCoV_N-positive Plasmid Control (IDT, Coralville, IA, USA). A cycle threshold (Ct) value of <40 was considered positive, based on CDC guidelines.

#### 2.3.2. Cellular RNA Extraction, Reverse Transcription, and Gene Expression

Cellular RNA isolation, reverse transcription (RT) into cDNA, as well as amplification and quantification through real-time qPCR were performed according to a standardized protocol [21]. Briefly, cells were washed in PBS and collected in RNAzol^®^ (TEL-TEST Inc., Friendswood, TX, USA) and RNA extraction was performed through the phenol-chloroform method. RNA was dissolved in RNase-free water and quantified by the Nanodrop 2000 Instrument (1 μL, Thermo Scientific, Waltham, MA, USA). One μg of RNA was purified from genomic DNA with RNase-free DNase (RQ1 DNase; Promega) and reverse transcribed into first-strand cDNA with Moloney murine leukemia virus reverse transcriptase along with random hexanucleotide primers, oligo dT, and dNTPs (Promega, Fitchburg, WI, USA). cDNA (200 ng) was amplified and quantified by real-time qPCR (CFX96 connect, Bio-Rad, Hercules, CA, USA) through SYBR Green PCR mix (Promega, Fitchburg, WI, USA), according to the following thermal profile: initial denaturation (95 °C, 15 min), denaturation (15 s at 95 °C × 40), annealing (1 min at 60 °C) and extension (20 s at 72 °C). Negative controls (distilled water, and RT-negative, total RNA sample), as well as positive controls (human cDNA), were included in each run. A Ct value of 40 or higher was considered negative. Melting curves were also analyzed for amplicon characterization. Results for gene expression analyses were calculated by the 2^−ΔΔCt^ equation and presented as the average of the relative expression units (in percentage) to an internal reference sample and normalized to the GAPDH housekeeping gene. Samples with GAPDH Ct values above 20 were excluded from the analysis. As an internal reference, a Mock-infected Control (Uninfected, vehicle-treated) with the best GAPDH Ct values (between 11 and 16) was chosen for each experiment, and thus set as 100%. The following genes were analyzed, D1 dopamine receptor (D1DR), D2 dopamine receptor (D2DR), L-Dopa Decarboxylase (DDC), dopamine transporter (DAT), Tyrosine Hydroxylase (TH), cAMP-dependent Protein Kinase A (PKA), cAMP Response Element-Binding protein (CREB), Mitogen-activated Protein Kinase 3 (MAPK3/ERK1), Glycogen Synthase Kinase 3-b (GSK3-β), Interferon A (IFNA1), Interferon B (IFNB), Interferon Regulatory Factor 3 (IRF3), Human myxovirus resistance protein 1 (MX-A), Toll-like Receptor 4 (TLR4), High-Mobility Group Box 1 (HMGB1), NLR family pyrin domain containing 3 Inflammasome (NLRP3), Interleukin 1b (IL-1b), Interleukin 6 (IL-6), Interleukin 4 (IL-4). Primers for DDC, PKA, MAPK3, CREB, GSK3-b, IFNA1, TRL4, NLRP3, IL-6, and HMGB1 genes were purchased as already optimized (PrimePCR, Bio-Rad, Segrate, Italy). Sequences of the remaining primers are listed in Supplementary Materials, Table S1.

### 2.4. Immunocytochemistry

Forty-eight hours post-infection (either Mock- or SARs-CoV-2 infection) cells were washed in PBS and fixed with 4% paraformaldehyde (PFA) for 10 min at room temperature (RT), followed by permeabilization with 0.1% Triton X-100 in PBS for 10 min at RT, blocking in 5% Bovine Serum Albumin (BSA) in PBS for 1 h at RT, and incubation for 1 h at RT with primary antibodies (Mouse anti-D2DR B-10: sc-5303, 1:200, Santa Cruz Biotechnology, Inc, Dallas, TX, USA; Rabbit anti-N Nucleocapsid SARS-CoV-2 Antibody NR-53791, 1:1000, obtained from Bei Resources, NIAID, NIH; Rabbit anti-D1DR bs-1007R, 1:200, Bioss, MA, USA; Mouse anti-S2 Spike SARS-CoV-2, 1:100, GeneTex, Alton Pkwy Irvine, CA, USA) prepared in 1% BSA-PBS. Cells were washed three times in PBS and incubated for 45 min at RT with secondary antibodies (Goat anti-mouse Alexa Fluor 488 (ab150113) or 647 (ab150115), or Goat anti-rabbit Alexa Fluor 488 (ab150077) or 647 (ab150079), 1:500, abcam, Cambridge, UK) prepared in 1% BSA-PBS. Negative controls were performed by omitting primary antibodies (Supplementary material, Figure S5). After three washes in PBS (5 min each), coverslips were carefully removed from the wells and mounted on Superfrost glass slides using a mounting medium with DAPI (Enzo Life Sciences, Milan, Italy). Confocal imaging was performed with a Leica TCS SP5 AOBS microscope system using a 40×/1.30 oil immersion objective (Leica Microsystems, Wetzlar, Germany). The images shown in the figures are representative results from independent experiments.

### 2.5. MTT and Trypan Blue Cell Viability Assays

CaLu-3 cells were seeded in 96-well plates (3 × 10^4^ per well) for 48 h and treated with the specific compounds following the same protocols detailed in Section 2.2. Cells were then either mock-infected or infected with SARS-CoV-2 as detailed in Section 2.2. After 48 h, cell viability was assessed by 3-(4,5-dimethylthiazol-2-yl)-2,5-diphenyltetrazolium bromide (MTT) method. Briefly, 30 μL of MTT (final concentration, 0.5 mg/mL) were added to each well under sterile conditions, and the 96-well plates were incubated for 4 h at 37 °C. Supernatants were removed, and dimethyl sulfoxide (100 µL/well) was added. The plates were then agitated on a plate shaker for 5 min. The absorbance of each well was measured at 490 nm with a Bio-Rad automated EIA analyzer (Bio-Rad Laboratories, Hercules, CA, USA). The viability of Control cells (Mock-infected, vehicle-treated) was considered 100%, while the other conditions were expressed as percentages of control.

For Trypan Blue exclusion assay, cells were incubated in Cell Dissociation Buffer 1X (Merck-Sigma, Milan, Italy, 500 μL for 12-well plates) for 10 min at 37 °C. Then, an equal volume of DMEM was added to the wells to stop the dissociation reaction. Cells were detached and collected in sterile 2 mL Eppendorf Tubes in a final volume of 1 mL. Cells were centrifuged at 8 min at 1200 rpm, the supernatant was carefully discarded, and cells were resuspended in 1 mL of fresh medium. Ten μL of cell suspension were incubated with 10 μL of 0.4% Trypan Blue (Merck-Sigma, Milan, Italy) for 10 min in 96-well plates. Ten microliters of the mix were loaded on chamber slides and counted with the T20 Automated Cell Counter (Bio-Rad Laboratories, Hercules, CA, USA).

### 2.6. Statistics

Results are presented as mean ± SEM from at least *n* = 3 independent experiments, each performed in triplicate. Results for viral replication are expressed as the absolute viral copy number, which was calculated from the SARS-CoV-2 N gene Ct values by referring to a standard curve from the quantified 2019-nCoV_N-positive Plasmid Control. Percent inhibition of viral replication is calculated through the following formula, “1-viral N gene copy number of SARS-CoV-2-infected treated cells/viral copy number of N gene SARS-CoV-2-infected untreated (Control) cells”. Results for gene expression analyses were calculated by the 2^−ΔΔCt^ equation and presented as the average of the relative expression units (expressed in percentage) to an internal reference sample and normalized to the GAPDH housekeeping gene. As an internal reference, one among the three biological replicates from Mock-infected Controls (uninfected, vehicle-treated cells) with the best GAPDH Ct values (between 11 and 16) was chosen for each experiment, and thus set as 100%. Statistical analyses were performed through the Student’s t-test or one-way ANOVA, as appropriate, with GraphPad Prism Version 5 (GraphPad Software, La Jolla, CA, USA). Results were considered significant for *p* values ≤ 0.05.

## 3. Results

### 3.1. DA in the Micromolar Range Reduces SARS-CoV-2 Replication in CaLu-3 Cells

**Figure 1:**
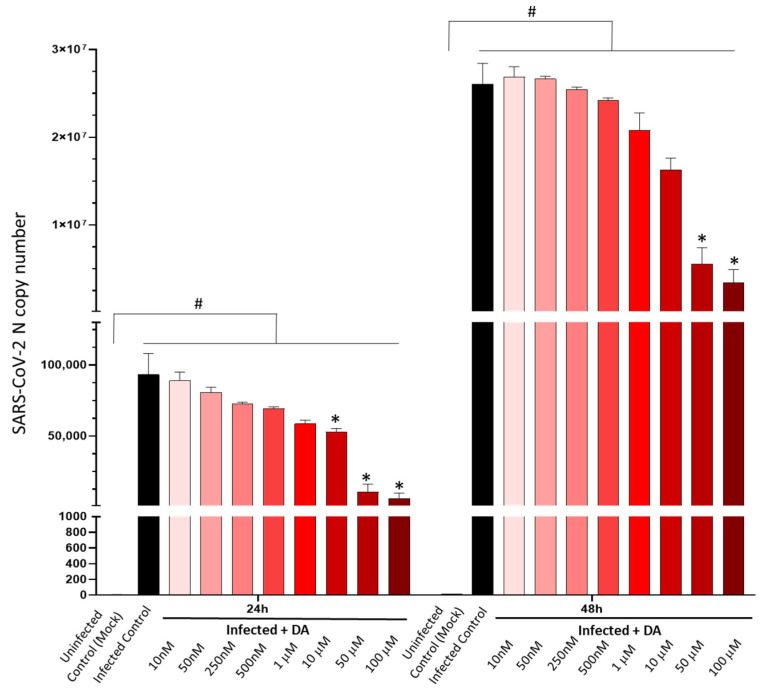
Dose- and time-related effects of dopamine in SARS-CoV-2 replication. Dopamine (DA) dose-dependently reduces SARS-CoV-2 replication at 24 and 48 h post-infection in CaLu-3 epithelial lung cells. Significant antiviral effects of DA occur in the micromolar range (10–100 μM). While the antiviral effects of the 10 μM DA dose lose significance at 48 h, DA at 50–100 μM doses produces a significant antiviral effect that persists at 48 h, exceeding 80% reduction in viral titers (SARS-CoV-2-N gene RNA levels). SARS-CoV-2 was not detected in the Uninfected Control (Mock). Results correspond to the absolute viral copy number of the SARS-CoV-2 N gene from cell supernatants that were quantified through a single-step, real-time, RT-qPCR by referring to a standard curve from RT-qPCR Ct values (IDT, Coralville, IA, USA). Results are presented as mean ± SEM from at least *n* = 3 independent experiments, each performed in triplicate. # *p* < 0.05 vs. Uninfected Control (Mock); * *p* < 0.05 vs. SARS-CoV-2-Infected Control.

Our data show that DA in the μM range (>1 μM) dose-dependently reduces SARS-CoV-2 replication in CaLu-3mhuman epithelial lung cells. While the 10 μM dose produced a moderate (nearly 50%), yet not significant, reduction in the amount of detectable viral RNA at 24 h, a remarkable anti-replicative effect (roughly 80%) persisting at 48 h was detected for the doses of 50 and 100 μM (Figure 1). Although the anti-SARS-CoV-2 effect of DA occurred at doses that surpass the physiological range, the cell viability assay ruled out any cytotoxic effects. In fact, DA was well-tolerated by CaLu-3 cells, inducing frank toxicity only above the 200 μM dose (Figure S1). Intriguingly, in Uninfected (Mock-infected) cells, DA between 1-to-50 μM doses produced a slight, yet significant proliferative effect, which was not observed in the presence of SARS-CoV-2 (Supplementary material, Figure S1). These data suggest that CaLu-3 cells well-tolerated high μM DA concentrations, ruling out any anti-proliferative effects related to the antiviral effects of DA observed in the range of 10–100 μM doses.

### 3.2. Dopamine Receptor Agonists Recapitulate While Dopamine Receptor Antagonists Reverse DA-Induced Inhibition of SARS-CoV-2 Replication

In light of the antiviral effect of DA at non-toxic μM DA range doses (10–100 μM), we administered D1DR and D2DR modulators either alone or in combination with DA to determine the contribution of D1DRs and D2DRs. For these assays, 50 μM DA was chosen as the lowest dose within the μM range that yielded significant anti-SARS-CoV-2 effects. In order to choose the appropriate doses of DR modulators, an MTT assay in both Mock- and SARS-CoV-2 infected cells was performed, which indicated the absence of any cytotoxic effects for any of these compounds at the dose of 10 μM, or their combination with DA 50 μM. In fact, only the D2DR antagonist at the dose of 50 μM produced a frank cytotoxic effect compared with both Uninfected (Mock) Control and SARS-CoV-2-infected Control (Supplementary material, Figure S2). To rule out any toxicities that are not reflected in cell proliferation, we also performed a Trypan Blue exclusion assay, which once again, confirmed the absence of cell toxicity for 50 μM DA or DR modulators at 10 μM, administered either alone or in combination with DA (Supplementary Figure S3). Thus, the dose of 50 μM and 10 μM was chosen for DA and D1/D2DR modulators, respectively, to assess their effects on SARS-CoV-2 replication at 48 h post-infection.

**Figure 2:**
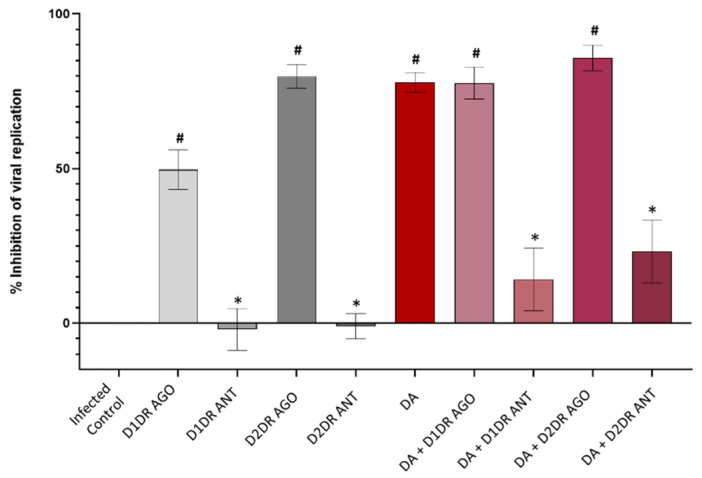
Effects of the D1 and D2 dopamine receptor agonists and antagonists in SARS-CoV-2 replication. D1DR and D2DR agonists (10 μM) reduce SARS-CoV-2 replication at 48 h post-infection, recapitulating the antiviral effect of DA (50 μM), which is instead reversed by D1DR and D2DR antagonists (10 μM). In detail, while the antiviral effect of the D1DR agonist alone is modest, reaching 50% of viral replication inhibition, the D2DR agonist either alone or in combination with DA, produces a robust antiviral effect (>80% inhibition of detectable viral RNA) which recapitulates and slightly surpasses that produced by DA alone. On the other hand, administration of either D1DR or D2DR antagonists alone favors SARS-CoV-2 replication, meanwhile occluding the antiviral effects of DA in the co-administration protocol. Values are expressed as percent (%) inhibition of viral replication, calculated as “1–SARS-CoV-2 N gene copy number of SARS-CoV-2-infected treated cells/SARS-CoV-2 N gene copy number of SARS-CoV-2-infected Control (untreated) cells”, with SARS-CoV-2 Infected Control corresponding to 0% Inhibition of viral replication. The absolute viral copy number of the SARS-CoV-2 N gene from cell supernatants was quantified through a single-step, real-time, RT-qPCR by referring to a standard curve from RT-qPCR Ct values (IDT, Coralville, IA, USA). Results are presented as mean ±SEM from at least *n* = 3 independent experiments, each performed in triplicate. # *p* < 0.05 vs. SARS-CoV-2-Infected Control; * *p* < 0.05 vs. DA 50 μM.

Both D1DR and D2DR agonists recapitulated, though at different magnitudes, the antiviral effect of DA, which was instead reversed by D1DR or D2DR antagonists (Figure 2). In detail, while the anti-replicative effect of the D1DR agonist was roughly comparable to that induced by the low-μM DA dose (10 μM, Figure 1), the D2DR agonist either alone or in combination with DA produced a robust antiviral effect which recapitulated that produced by DA at 50 μM dose (Figure 2). This suggests that SARS-CoV-2 is more sensitive to the effects of D2DR compared with D1DR agonists at comparable doses.

### 3.3. The Antiviral Effect of DA Is Associated with Downregulation of D2DRs

**Figure 3:**
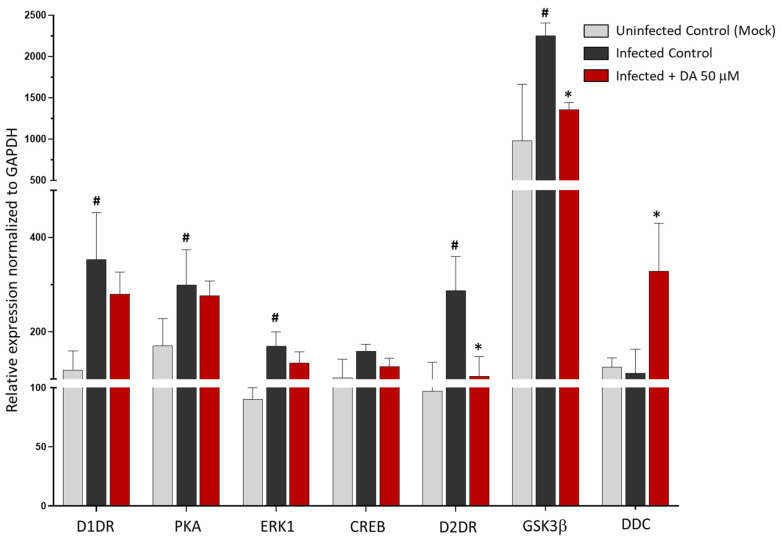
Dopamine-induced changes in the expression of D1DR, D2DR, downstream effector genes, and DA synthetic enzyme DDC. Compared with Uninfected Control (Mock), SARS-CoV-2 significantly increases the expression of D1DR, D2DR, and respective downstream effectors PKA, ERK1, and GSK3β at 48 h post-infection. Compared with the SARS-CoV-2-Infected Control, 50 μM DA administration to SARS-CoV-2-Infected cells markedly reduces the expression of D2DR and the downstream kinase GSK3-β while increasing DDC expression. On the other hand, DA does not significantly affect the expression of D1DR or downstream effectors. mRNA quantification was performed through real-time qPCR and calculated by the 2^−ΔΔCt^ equation. Results are presented as mean ± SEM from at least *n* = 3 independent experiments, each performed in triplicate. # *p* < 0.05 vs. Uninfected Control (Mock), * *p* < 0.05 vs. SARS-CoV-2 Infected Control.

Since DA and DR agonists are known to induce a phenomenon of receptor desensitization that eventually leads to the downregulation of DRs, we sought to determine whether the antiviral effects of these compounds are associated with any alterations in DRs’ expression. Remarkably, following SARS-CoV-2 infection, the expression of D1DRs and D2DRs increased by at least 2-fold compared with Uninfected Control (Mock). Noteworthy, compared with SARS-CoV-2 Infected Control, DA dramatically downregulated the expression of D2DR along with the downstream kinase GSK3-β, while it did not significantly affect the expression of either D1DR or downstream effectors such as PKA, ERK1, and CREB (Figure 3). At the same time, DA was able to rescue the expression of DDC (Figure 3), though the underlying mechanism remains to be clarified. The lack of evident effects of 50 μM DA upon D1DR expression is in line with the 10 to 100-fold lower affinity for D1-like compared with D2-like DRs (low vs. moderate μM DA doses, respectively) [11]. On the other hand, the remarkable decrease in both D2DR and the downstream effector GSK3-β expression suggests that the anti-SARS-CoV-2 effects of DA are related to the downregulation of D2DRs.

**Figure 4:**
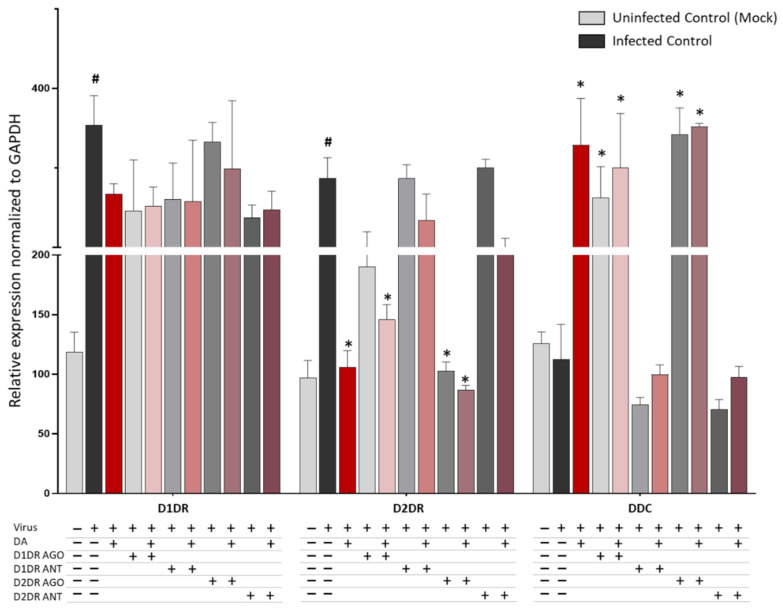
D1 and D2 receptor agonist/antagonist-induced changes in the expression of D1DR, D2DR, and DDC. Similar to DA 50 μM, administration of DR agonists (10 μM) to SARS-CoV-2-Infected cells, either alone or in combination with DA, decreases the expression of D2DR while increasing the expression of DDC compared with SARS-CoV-2-Infected Control at 48 h post-infection. The most robust effects are produced by D2DR agonists either alone or in combination with DA. mRNA quantification was performed through real-time qPCR and calculated by the 2^−ΔΔCt^ equation. Results are presented as mean ± SEM from at least *n* = 3 independent experiments, each performed in triplicate. # *p* < 0.05 vs. Uninfected Control (Mock), * *p* < 0.05 vs. SARS-CoV-2-Infected Control.

It is remarkable that DR agonists, and mostly the D2DR agonist, produced a similar scenario in the expression levels of D1DR, D2DR, and DDC within SARS-CoV-2-infected cells. While neither the D1DR nor the D2DR agonist altered the expression of D1DRs, either compound decreased, though at different magnitudes, the expression of D2DRs, meanwhile augmenting the expression DDC (Figure 4). Indeed, the D2DR agonist produced a robust reduction of D2DR expression, which was slightly potentiated by co-administration of DA. Instead, the D2DR antagonist produced opposite effects, suggesting that agonist-induced downregulation rather than the mere blockade of D2DR activity, is key to halting SARS-CoV-2 replication.

**Figure 5:**
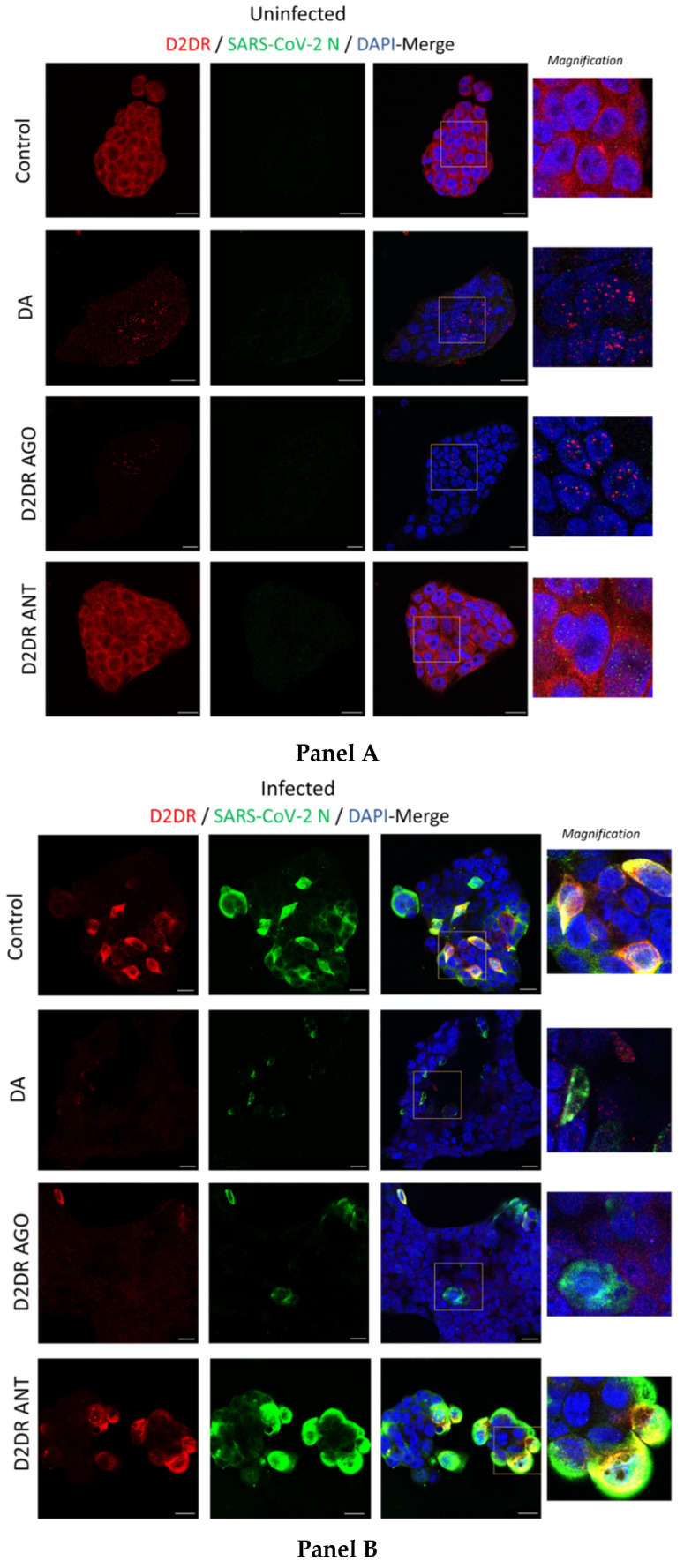
Representative images of combined immunofluorescence for D2DR and SARS-CoV-2 N protein. The anti-replicative effects of dopamine (DA, 50 μM) and D2DR agonist quinpirole (10 μM) at 48 h post-infection are associated with downregulation of D2DRs in either Uninfected (Mock, **Panel A**) and SARS-CoV-2-Infected cells (**Panel B**). Compared with Uninfected Control cells (**Panel A**, Control), SARS-CoV-2 produces a dramatic clustering of D2DRs both at the plasma membrane and adjacent intracellular compartments, where an impressive co-localization with the SARS-CoV-2-N protein occurs (**Panel B**, Control). On the other hand, administration of DA, similar to the D2DR agonist, dramatically reduces D2DR staining leading to the formation of intracellular red puncta in both Uninfected (DA, and D2DR Ago, **Panel A**), and SARS-CoV-2-Infected (DA, and D2DR Ago, **Panel B**) cells. Such a scenario is not observed following D2DR antagonist administration in either Uninfected (Mock), or SARS-CoV-2-infected cells (D2DR Ant, **Panel A** and **Panel B**, respectively). In SARS-CoV-2 infected cells, the reduction in SARS-CoV-2-N protein staining that is produced by either DA or the D2DR agonist (**Panel B**) goes along with a dramatic reduction of D2DR immunostaining and a disruption of their co-localization. This is instead occluded by the administration of the D2DR antagonist, whereby the co-localization between D2DR and N-SARS-CoV-2 protein is magnified, as evident by the occurrence of both wide, overlapping areas and large intracellular puncta (D2DR Ant, **Panel B**). Cells were incubated with primary antibodies (Mouse anti-D2DR B-10: sc-5303, 1:200, Santa Cruz Biotechnology, Inc, Dallas, USA; Rabbit anti-N Nucleocapsid SARS-CoV-2 Antibody NR-53791, 1:1000, obtained from Bei Resources, NIAID, NIH), and with secondary antibodies [Goat anti-mouse Alexa Fluor 647 (ab150115), and Goat anti-rabbit Alexa Fluor 488 (ab150077), 1:500, abcam, Cambridge, UK]. Coverslips were mounted on Superfrost glass slides using a mounting medium with DAPI (Enzo Life Sciences, Milan, Italy). Confocal imaging was performed with a Leica TCS SP5 AOBS microscope system using a 40X/1.30 oil immersion objective (Leica Microsystems, Wetzlar, Germany). The images shown in the figures are representative results from independent experiments. Bars correspond to 20 μM.

The downregulation of D2DRs that is observed following administration of either DA or the D2DR agonist may be related to four, non-necessarily mutually-exclusive events [22,23,24], namely (i) inhibition of mRNA receptor transcription, (ii) reduction in the receptor mRNA steady-state levels, (iii) post-transcriptional agonist-mediated degradation of mRNA and iv) post-translational receptor degradation following agonist-induced receptor internalization and sequestration into proteolytic organelles. In order to assess whether SARS-CoV-2 and treatment with DA or DR agonists alter DRs at protein levels, we performed double immunostaining at fluorescence microscopy for DRs and SARS-CoV-2 proteins. Impressively, while D2DR staining in Uninfected control (Mock) showed a diffuse pattern with enhanced immune reactivity at the level of the plasma membrane (Figure 5, Panel A), SARS-CoV-2 produced a dramatic clustering of D2DRs at the plasma membrane and adjacent intracellular compartments, where a clear-cut co-localization with the viral Nucleocapsid (N) protein emerged, suggesting that SARS-CoV-2 may exploit D2DR for host cell invasion and replication (Figure 5, Panel B). In support of this hypothesis, administration of DA, similar to the D2DR agonist, dramatically disrupted such a co-localization by reducing both SARS-CoV-2 infection and D2DR abundance. Such an effect was instead reversed by the D2DR antagonist (Figure 5, Panel B). A dramatic reduction of D2DR staining, similar to that observed in SARS-CoV-2 infected cells, was also evident in Uninfected (Mock) cells following administration of DA or D2DR agonist (Figure 5, Panel A). As mentioned above, this might be due to either transcriptional (inhibition of D2DR mRNA transcription), post-transcriptional (degradation of D2DR mRNA and subsequent reduction of protein synthesis), or post-translational events (agonist-dependent receptor internalization, and degradation). However, these events are not necessarily mutually exclusive. Indeed, while DA and the D2DR agonist produced a rough 65% reduction in D2DR mRNA levels in SARS-CoV-2 infected cells, the downregulation at protein D2DR levels was almost abolished by these compounds in both Mock- and SARS-CoV-2 infected cells (Figure 5A,B, respectively). In line with recent studies, this suggests the potential involvement of agonist-related post-translational events fostering degradation of D2DRs [25], which remains to be confirmed in CaLu-3 cells specifically through tailored methodological approaches targeting endocytic and degradation pathways.

In summary, these data cast the hypothesis that agonist-induced D2DR activation and subsequent downregulation interfere with SARS-CoV-2 replication either by reducing the amount of plasma membrane receptors available for SARS-CoV-2 entry and/or by fostering degradation of the internalized D2DR-SARS-CoV-2 complex, which remains to be confirmed.

At first glance, no clear-cut correlation emerged between D1DR and SARS-CoV-2 staining patterns instead (Supplementary material, Figure S4). At a closer look, the D1DR agonist enhanced D1DR staining, especially at the intracellular level, while the D1DR antagonist produced an opposite effect, suggesting a potentially protective role for D1DR. However, it appears hazardous either to draw any sound conclusions on the role of D1DRs, as it emerged for D2DR, or hypothesize a phenomenon of agonist induced D1DR internalization and downregulation. In light of these considerations, we focused on D2DRs as a major target underlying the robust antiviral effects of DA and D2DR agonists in order to further examine potential links with innate immunity pathways in the frame of SARS-CoV-2 infection.

### 3.4. The Antiviral Effect of DA and D2DR Agonist Is Associated with Upregulation of Type-I Interferons and Downregulation of Pro-Inflammatory Mediators

**Figure 6:**
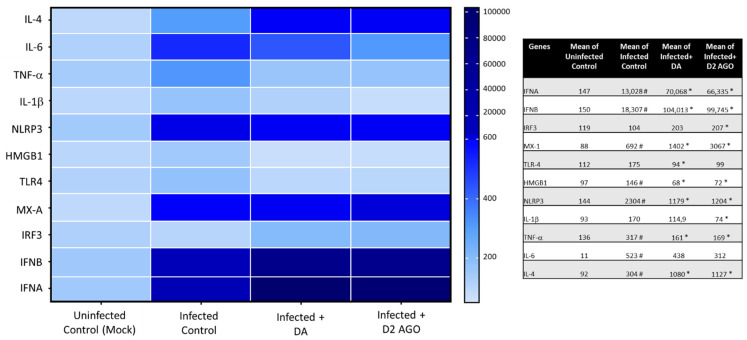
Transcriptional analysis of innate immunity and pro-inflammatory pathways modulated by DA and the D2DR agonist. Within SARS-CoV-2-Infected cells, DA and the D2DR agonist produce an overlapping transcriptional profile that includes potentiation of Type-I IFN response genes and downregulation of pro-inflammatory pathways and cytokines. In detail, compared with Uninfected Control (Mock), SARS-CoV-2 (Infected Control) increases the expression of IFNA, IFNB, MX-1, HMGB1/TLR4, NLRP3/IL-1β, as well as IL-6 and IL-4. Instead, administration of DA to SARS-CoV-2 infected cells, similar to the D2DR agonist, markedly upregulates the expression of IFNA, IFNB, MX-1, and to a lesser extent IRF3 compared with Infected Control, suggesting potentiation of innate immune antiviral pathways. At the same time, DA, and the D2DR agonist downregulate HMGB1/TLR4, NLRP3/IL-1β, and IL-6 while increasing IL-4 mRNA levels, suggesting the induction of an anti-inflammatory profile. mRNA quantification was performed through real-time qPCR and calculated by the 2^−ΔΔCt^ equation. Results shown on the heatmap correspond to the mean ± SEM from at least *n* = 3 independent experiments, each performed in triplicate. Quantified mean values are shown in the table. # *p* < 0.05 vs. Uninfected Control (Mock), * *p* < 0.05 vs. Infected (SARS-CoV-2) Control.

In search of molecular pathways associated with the antiviral effect of DA and D2DR agonists, we performed a transcriptional analysis of a wide variety of genes implicated in innate immunity, including IFN signaling pathways, such as Type-I IFNs (IFNA, IFNB), IFN-stimulating genes (ISGs, IRF3) and antiviral effectors (MX-A), along with inflammatory cytokines and receptors which are known to be involved SARS-CoV-2 infection. These include pro-inflammatory mediators IL-6, TNF-α, NLRP3/IL-1β, TLR4/HMGB1 pathway, or anti-inflammatory mediators such as IL-4. Remarkably, within SARS-CoV-2 Infected cells, both DA and the D2DR agonist produced a similar transcriptional profile which was associated with a dramatic augmentation of Type-I IFNs and MX-A, while IRF3 was only moderately enhanced. A remarkable reduction in the levels of pro-inflammatory mediators NLRP3/IL-1β, and TLR4/HMGB1 pathways, as well as TNF-α and IL-6, was also detected, while IL-4 was instead augmented by both compounds (Figure 6). Although these data need to be confirmed at protein levels, they suggest that the antiviral effects of DA and D2DR agonists are bound to the enhancement of Type-I IFNs, along with downregulation of NLRP3 inflammasome and Danger Associated Molecular Pattern (DAMP) signaling pathways.

### 3.5. Type-I Interferons Suppress SARS-CoV-2 Replication, Which Is Associated with Downregulation of D2DRs and the Rescue of DA Synthetic Enzymes

**Figure 7:**
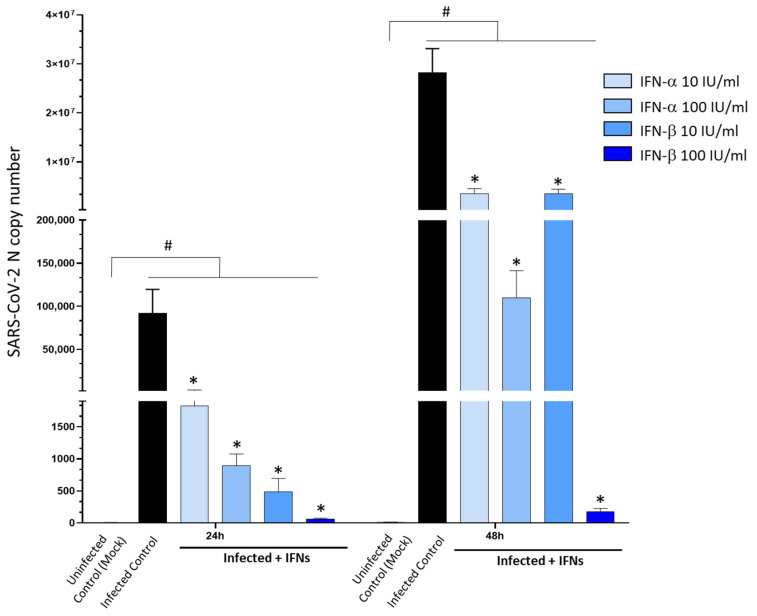
Dose- and time-related effects of Type-I Interferons in SARS-CoV-2 replication. Administration of Type-I Interferons (IFN-α, β, 10 and 100 IU/mL) dose-dependently inhibits SARS-CoV-2 replication in CaLu-3 cells. The strongest antiviral effect is observed for 100 IU/mL IFN-β, leading to nearly 100% inhibition of viral replication at 24 and 48 h. SARS-CoV-2 was not detected in the Uninfected Control (Mock). Results correspond to the absolute viral copy number of the SARS-CoV-2 N gene from cell supernatants that were quantified through a single-step, real-time, RT-qPCR by referring to a standard curve from RT-qPCR Ct values (IDT, Coralville, IA, USA). Results are presented as mean ± SEM from at least *n* = 3 independent experiments, each performed in triplicate. # *p* < 0.05 vs. Uninfected Control (Mock); * *p* < 0.05 vs. Infected Control.

Eventually, we sought to determine the molecular mechanisms bridging Type-I Interferon (IFN) response and DA pathways in SARS-CoV-2 infection. In line with previous evidence, administration of Type-I IFNs, especially IFN-β, dramatically reduced SARS-CoV-2 replication (Figure 7), leading to nearly 100% inhibition at the dose of 100 IU at 48 h. As shown by the MTT assay, neither IFN-α nor IFN-β at the dose of 100 IU/mL produced any significant alterations in cell viability in either Mock- or SARS-CoV-2-infected cells (Supplementary Figure S6).

**Figure 8:**
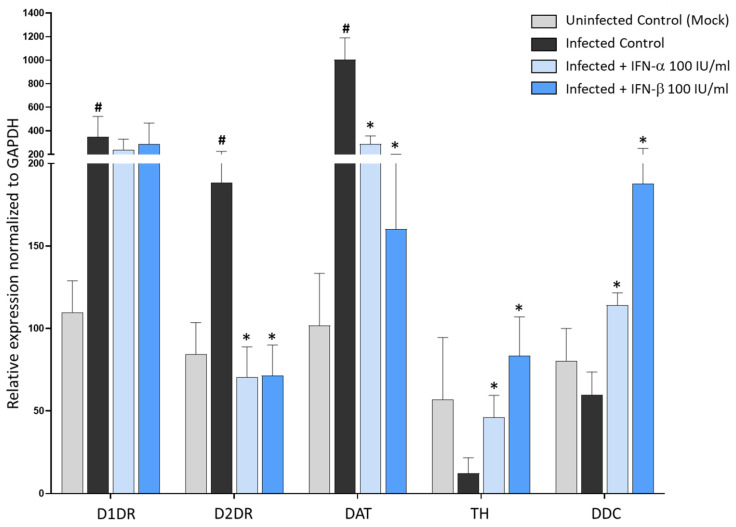
Type-I Interferons reverse SARS-CoV-2 induced changes in the expression of DA-related genes. Compared with Uninfected Control (Mock), SARS-CoV-2 (Infected Control) at 48 h post-infection potentiates the expression of D1DR, D2DR, and DAT meanwhile downregulating TH and DDC. Treatment of SARS-CoV-2-Infected cells with Type-I Interferons (IFN-α, -β, 100 IU/mL) reduces D2DR and DAT expression while potentiating TH and DDC expression in CaLu-3 cells. mRNA quantification was performed through real-time qPCR and calculated by the 2^−ΔΔCt^ equation. Results are presented as mean ± SEM from at least *n* = 3 independent experiments, each performed in triplicate. # *p* < 0.05 vs. Uninfected Control (Mock), * *p* < 0.05 vs. SARS-CoV-2-Infected Control (SARS-CoV-2).

Thus, we chose the dose of 100 IU/mL for both IFN-α and -β to examine their transcriptional effects on the DA system’s targets. Remarkably, in SARS-CoV-2 infected cells, both IFNs converged in strongly downregulating the expression of D2DRs, while D1DRs were not significantly affected, strengthening the major role of D2DR in acting as a bridge in the anti-SARS-CoV-2 effects of DA and Type-I IFNs (Figure 8). Noteworthy, when examining the effects of IFNs upon DA synthetic and transport pathways, we disclosed two important findings related to SARS-CoV-2-induced alterations at the level of the DA system within human epithelial lung cells. Firstly, besides D1DR and D2DR, within CaLu-3 cells, we could detect for the first time the mRNA expression of DA synthetic enzymes TH, and DDC, and the DA transporter DAT. Remarkably, while SARS-CoV-2 infection strongly induced the expression of the DAT along with D1DR and D2DR, it suppressed the expression of TH while slightly downregulating DDC compared with Uninfected Control (Figure 8). The expression of these genes in CaLu-3 cells is also supported by their representative melting curves (Supplementary Figure S7). In this frame, it should be pointed out that, contrarily to D1DR, D2DR, and DDC, TH was only moderately expressed at baseline (Uninfected Control). Instead, DAT was very weakly expressed at baseline, while it was increased by nearly 10-fold following SARS-CoV-2 infection (Figure 8), as also witnessed by the melting peaks (Supplementary Figure S7). Thus, it is likely that the weaker baseline expression of TH and DAT might contribute to magnifying the effects of SARS-CoV-2 upon TH and DAT compared with DRs which are increased by 2-fold instead. This notwithstanding, our data suggest that SARS-CoV-2 infection in the lung epithelium produces dramatic alterations at the level of DA pathways, and CaLu-3 cells might represent a suitable model to study these effects. The second remarkable point is that SARS-CoV-2-induced alterations at the level of DA genes here examined were indeed reversed by Type-I IFNs (Figure 8). In fact, while downregulating D2DR and DAT, IFNs rescued TH and DDC expression. These data indicate broad effects of both SARS-CoV-2 and Type-I IFNs in DA-related pathways, including DA synthesis, and intracellular DA re-uptake, which in turn, are a key to determining the extracellular availability, and subsequent effects of DA upon D1DR and D2DR. In fact, the increase in DAT expression following SARS-CoV-2 infection as compared with Uninfected (Mock) controls, might provide a rationale for the μM DA dosage that is required to produce anti-SARS-CoV-2 effects within these cells.

### 3.6. A549-hACE2 Lack D2DRs and Are Less Susceptible to Both SARS-CoV-2 Infection and Type-IFN Treatment

**Figure 9:**
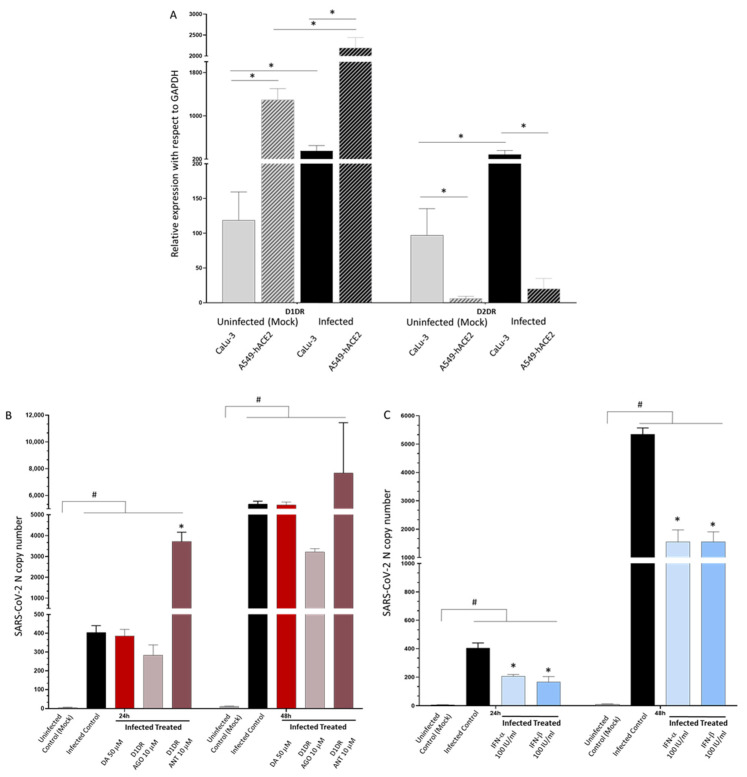
The lack of D2DR expression in A549-hACE2 cells is associated with refractoriness to DA administration and low susceptibility to both SARS-CoV-2 infection and Type-I IFN treatment. (**A**) Relative expressions of D1DR and D2DR in A549-hACE2 compared with Calu-3 cells in either Mock-infected and SARS-CoV-2 infected cells at 48 h post-infection. At baseline (Uninfected, Mock), D1DR expression is higher in A549-hACE2 cells while D2DR is almost undetectable compared with CaLu-3 cells. SARS-CoV-2 (Infected Control) increases D1DR expression by 2-fold and 1.5-fold compared with Uninfected Control in CaLu-3, and A549-hACE2 cells, respectively, while a 2-fold increase in D2DR expression is observed only in CaLu-3 cells. In fact, even following SARS-CoV-2 infection, D2DR expression remains almost undetectable in A549-ACE2 cells. mRNA quantification was performed through real-time qPCR and calculated by the 2^−ΔΔCt^ equation. Results are presented as mean ± SEM from at least *n* = 3 independent experiments, each performed in triplicate. * *p* < 0.05. (**B**) Effect of DA and DR modulators on SARS-CoV-2 replication within A549-hACE2 cells. A549-hACE2 cells are refractory to the effects of DA 50 μM, while the D1DR agonist 10 μM decreases SARS-CoV-2 replication by roughly 30 and 40%, at 24 and 48 h, respectively. On the other hand, the D1DR antagonist 10 μM significantly favors SARS-CoV-2 replication at both 24 and 48 h. SARS-CoV-2 was not detected in the Uninfected Control (Mock). Results correspond to the absolute viral copy number of the SARS-CoV-2 N gene from cell supernatants that were quantified through a single-step, real-time, RT-qPCR by referring to a standard curve from RT-qPCR Ct values (IDT, Coralville, IA, USA). Results are presented as mean ± SEM from at least *n* = 3 independent experiments, each performed in triplicate. # *p* < 0.05 vs. Uninfected Control (Mock); * *p* < 0.05 vs. Infected Control (SARS-CoV-2). (**C**) Effects of Type-I Interferons on SARS-CoV-2 replication within A549-hACE2 cells. In A549-hACE2 cells, Type-I IFNs produce a rough 50 and 70% inhibition of SARS-CoV-2 replication at 24 and 48 h, respectively. SARS-CoV-2 was not detected in the Uninfected Control (Mock). Results correspond to the absolute viral copy number of the SARS-CoV-2 N gene from cell supernatants that were quantified through a single-step, real-time, RT-qPCR by referring to a standard curve from RT-qPCR Ct values (IDT, Coralville, IA, USA). Results are presented as mean ± SEM from at least *n* = 3 independent experiments, each performed in triplicate. # *p* < 0.05 vs. Uninfected Control (Mock); * *p* < 0.05 vs. Infected Control (SARS-CoV-2).

When investigating the potential antiviral effects of DA in other cell lines, we made a serendipitous finding which strengthened the hypothesis of a correlation among D2DR expression, the effects of DA, SARS-CoV-2 susceptibility, and Type-I IFNs response. Indeed, compared with CaLu-3 cells, the expression of D2DR in lung A549-hACE2 was barely detectable, while D1DR was instead expressed to a higher extent (Figure 9A). Albeit the exogenous expression of ACE2, such cells are less susceptible to SARS-CoV-2 infection at comparable MOI with respect to CaLu-3 cells, and remarkably, they are also less sensitive to administration of either DA (50 μM, Figure 9B) or Type-I IFNs (100 UI/mL, Figure 9C). In fact, contrarily to CaLu-3 cells whereby IFNs almost completely abolish viral replication, in A549-hACE2 they produce a rough 50 and 70% inhibition of SARS-CoV-2 replication at 24 and 48 h, respectively (Figure 9C). Again, SARS-CoV-2-infected A549-hACE2 cells were refractory to the effects of DA, strengthening the hypothesis that DA acts mainly through D2DR; instead, the D1DR agonist exerted a modest anti-replicative effect, whilst the D1DR antagonist significantly favored SARS-CoV-2 replication, suggesting a potentially protective role for D1DRs (Figure 9B). This strengthens the hypothesis that D2DRs play a major role in influencing the susceptibility to both SARS-CoV-2 infection and Type-I IFNs response.

## 4. Discussion and Conclusions

Alterations of the systemic or local DA system occur in both experimental models and patients with various inflammatory diseases [9,10,26]. Beyond the CNS, DA synthetic enzymes as well as DRs are expressed in a variety of organs and immune cell subpopulations, where they modulate pathological processes by interacting with a plethora of inflammatory mediators and pathways [6,10,27]. For instance, DA acting through DRs modulates the innate immune response to viral infections including HIV, Ebola Virus, and the Japanese Encephalitis Virus [27,28,29]. The latter also exploits DA transduction pathways to increase neuronal susceptibility to infection.

In the lung, the major site of SARS-CoV-2 infection, DRs are expressed by alveolar epithelial cells, lung macrophages, and pulmonary terminal nerves. Thus, it is not surprising that DA in the lungs modulates a variety of physiological functions, including ventilation, pulmonary circulation, bronchial diameter, and water clearance, meanwhile shaping immune responses [12,13,27]. Expression of D1DR and D2DR has been already detected in human epithelial lung cell lines CaLu-3 and CaLu-6 [30,31]. To our knowledge, none of the other DA-related targets (DDC, TH, DAT) has been ever investigated in CaLu cells specifically, though their expressions are reported in the lungs from either human subjects or experimental animals [13,26,30,32,33,34]. Here, within CaLu-3 cells, we could detect the expression of both D1DR and D2DR, as well as DA synthetic enzymes, which makes them a suitable model to investigate SARS-CoV-2-induced alterations in the DA system. Since the DA system in the lungs is maximally developed at birth to cope with lung water clearance [13], it is likely that CaLu-3 cells might have retained some neuroepithelial/neuroendocrine properties due to their tumoral, stemness-like properties.

Although several hypotheses have been put forward on the role of DA in the frame of COVID-19, especially in relation to parkinsonism [1,4,35], to our knowledge this is the first report documenting a beneficial role of DA in SARS-CoV-2 infection within human epithelial lung cells. Although both D1DR and D2DR were enhanced following SARS-CoV-2 infection, downregulation of D2DR but D1DR emerged as the convergent mechanism underlying the anti-SARS-CoV-2 effects produced by DA, DR agonists, as well as Type-I IFNs.

It should be pointed out that the doses of DA and DR modulators that display significant effects in SARS-CoV-2 replication in the present study are likely to surpass the physiological concentrations detected in the blood and periphery [6]. While DA concentrations in synapses may peak in the μM range [36], DA concentrations detected in the periphery are extremely variable among different organs and species, as extensively reviewed elsewhere [6]. However, high DA concentrations are detected in the rodent kidney and ruminant lungs, ranging from 10^−4^ M to 10^−8^ M, and from 10^−5^ M to 10^−8^ M, respectively [6]. In the human lungs, DA concentrations are above those in the adrenal glands, the main peripheral source of DA, though data from human subjects remain scarce [6,13]. In line with our results, the antiviral activity of other indirect DA agonists (amantadine) has been recently reported to occur at μM concentrations (between 83 and 119 µM) in the frame of SARs-CoV-2 infection in vitro [17].

Several potential explanations for this phenomenon exist. Firstly, the presence of the DA transporter DAT, which is remarkably increased here following SARS-CoV-2 infection in CaLu-3 cells, may contribute to influencing the extracellular availability of DA to exert its effects upon DRs.

Again, DA and D1/D2DR agonist doses in the micromolar range (10–100 μM) may be required to produce specific effects that might be relevant for SARS-CoV-2 infection as well. These include inhibition of systemic inflammation through downregulation of pro-inflammatory cytokines and NLRP3 as documented within various cell types, as well as liquid lung clearance through modulation of ion channel activity, as documented in alveolar and bronchial epithelial cells both in vitro and in vivo [5,13,32,37,38]. The high, yet non-toxic DA concentrations needed for such effects, similar to those required for inhibition of SARS-CoV-2 replication in our study, might be due to the instability of DA [5]. In fact, in both cases, these beneficial effects are observed when a sub-chronic, rather than acute DA administration protocol in the μM range is employed.

Eventually, DA as well as DR agonists at saturating μM doses may be required to induce long-lasting desensitization and internalization of DRs. Indeed, highlighting the fact that agonist-induced GPCR internalization is both a dose and time-dependent, and cell-specific event [24,39,40], a plethora of studies documented receptor internalization and downregulation following administration of DA and DR modulators in the μM range [40,41,42,43,44]. This might explain why in the present study DA produces a remarkable antiviral effect overlapping with that yielded by the D2DR agonist and Type-I IFNs only at doses above 10 μM. Furthermore, D2DRs possess 10- to 100-fold greater affinity to DA compared with D1DRs, which supports the hypothesis that the robust antiviral effect that occurs above the 10 μM DA dose, is mostly related to agonist-induced downregulation of D2DR. Again, downregulation of the downstream D2DR effector GSK3-β supports this hypothesis. Indeed, downregulation or inhibition of GSK3-β was recently shown to reduce SARS-CoV-2 replication [45], strengthening the potential key role of D2DRs in SARS-CoV-2 infection.

Since D2DR antagonists occluded the antiviral effect of DA, it is likely that agonist-induced downregulation rather than the mere blockade of D2DR activity is key to halting SARS-CoV-2 replication. This is in line with both pioneer and recent studies indicating that agonist binding to GPCRs triggers transduction cascades which foster receptor signaling termination and their downregulation at both mRNA and protein levels [22,23,24,25,39,40,43,44,46]. In the case of DA specifically, the latter event was recently shown to involve agonist-induced activation of degradation pathways, which is occluded by D2DR antagonists instead [25]. Thus, asides from, or along with agonist-induced mechanisms affecting D2DR mRNA transcription [23], endosomal/autophagy pathways are triggered by DA or D2DR agonists, with D2-like DRs being more sensitive to their endogenous ligand-induced degradation compared with D1-like DRs [25], which is in line with our results.

Nevertheless, the role of D1DRs remains unclear. In this frame, it is worth mentioning that D1DRs feature different internalization fates and recycling dynamics compared with D2DRs [25,47]. While agonist-induced internalization of D2DR is rapid and may last from hours to days involving degradation of endocytosed receptors, D1DR internalization is an extremely rapid event, with D1DRs skewing degradation pathways. This is confirmed by their ability to signal from endosomal intermediates [47]. Both the narrow timeframe of D1DR internalization and their rapid recycling dynamics, incompatibly with the wide timeframe which is necessary for effective SARS-CoV-2 replication, are expected to impede the ready detection of changes related to D1DR receptor abundance following agonist treatments. Since D1DR antagonists could reverse the anti-replicative effects of DA while weakening D1DR immunostaining in CaLu-3 cells, it is likely that the increase observed at D1DR levels following SARS-CoV-2 infection represents a compensatory cytoprotective response. Again, the pro-viral effect of the D1DR antagonist in A549-hACE2 supports their protective role. Nonetheless, further investigations through tailored methodological approaches, such as radioligand binding assay, are needed to clarify the specific involvement of DRs in SARS-CoV-2 infection. In this frame, we cannot exclude that besides specific effects on D2DRs, the antiviral effects of DA and DR modulators may be bound, at least in part, to adenosine, norepinephrine, and serotonin pathways, which remain to be investigated as well.

Although we could not detect significant changes at the level of DDC mRNA following SARS-CoV-2 infection, the antiviral effect of either DA, DR agonists, and Type-I IFNs was associated with a remarkable increase in TH and DDC expression. This indirectly suggests that SARS-CoV-2 might affect DA synthesis, as previously documented in non-neuronal cells [2], while Type-I IFNs are able to reverse such an effect. Intriguingly, DDC expression is decreased in the lungs of subjects with idiopathic pulmonary fibrosis [26], which in turn, is associated with both COVID-19 susceptibility and severity, suggesting a potentially protective role for DA signaling. Again, in this frame, it is likely that D2DR downregulation represents the convergent mechanism through which Type-I IFNs rescue TH and DDC expression, and potentially, DA synthesis. In neuronal cells within the ventral tegmental area, D2DR auto-receptors are known to exert an inhibitory tone on DA synthesis and release while accelerating DA reuptake through the DAT [48]. Following the release, DA activates these auto-receptors to decrease the probability of an excess DA release due to subsequent presynaptic stimulation, which is a rapid event generally lasting up to several seconds. Such an event is followed by a mechanism involving auto-receptor desensitization and downregulation, a form of plasticity that promotes burst firing in DA neurons [48]. Thus, D2DR downregulation, as observed in the present study following sub-chronic exposure to DA, DA agonist, or IFNs, is expected to rescue DA synthetic pathways, which remains to be confirmed in CaLu-3 cells.

Intriguingly, administration of Type-I IFNs, besides upregulating DDC and TH enzymes that were downregulated by SARS-CoV-2, was able to downregulate the expression of the DAT, which was instead upregulated by SARS-CoV-2. This suggests that, while increasing DA synthesis, IFNs may increase the availability of extracellular DA to act via DRs. In turn, DA acting on D2DRs may trigger a rapid transduction cascade which fosters induction of IFN-stimulating genes (ISGs) and transcription of Type-I IFNs, followed by downregulation of D2DRs. These events may eventually contribute to hindering SARS-CoV-2 infection while restraining hyper-inflammation to limit tissue damage. In fact, DA, similar to the D2DR agonist was able to downregulate key pro-inflammatory mediators that are also implicated in COVID-19 severity, including IL-6, TNF-α, and NLRP3/IL-1b, TRL4/HMGB1 pathways. Besides activation of TLR4 by SARS-CoV-2-released DAMPs such as HMGB1, TLR4 has been predicted to own strong binding affinity to the SARS-CoV-2 spike protein directly. Thus, COVID-19 severity is bound to an enhancement of TLR4-mediated inflammatory signaling, including the release of TNF-α and other pro-inflammatory cytokines [49,50,51,52]. These events also instigate NLRP3 inflammasome activation, eventually causing an intensely exacerbated immune response due to cytokine storm in the host’s lungs, which may lead to multiple organ failure [53]. As such, targeting the TLR4/HMGB1 and NLRP3 inflammasome/IL-1β axis may be beneficial in the frame of COVID-19 [50,54]. Our results are in line with previous evidence documenting that DA inhibits NLRP3 inflammasome by increasing its ubiquitination and degradation, which has been attributed to either D1DR or D2DR pathways [5,55].

Adding to previous evidence on the beneficial role of Type-I IFNs in SARS-CoV-2 replication [56,57,58,59,60] here we document that such effects are bound to the rescue of alterations in DA pathways. The link between Type-I IFNs and DA system integrity is supported by evidence that the lack of IFN-β signaling in murine models causes defective DA signaling, reduction of DA neurons, and movement disorders reminiscent of PD, which are associated with deficits in degradation pathways [61]. This underlines the need for further studies investigating the role of the endocytic/autophagy-lysosomal pathways in the mechanisms bridging D2DR and IFN responses, in as much as they are modulated downstream of DA receptors and modulate in turn D2DR internalization, meanwhile being involved in Type-I IFN response and SARS-CoV-2 replication [7,25,62].

It is remarkable that the scenario produced by SARS-CoV-2 at the level of DA pathways documented in the present study is reminiscent of PD pathogenesis, whereby the loss of DA weakens the direct pathway mediated by D1DRs while strengthening the indirect pathway mediated by D2DRs. This eventually leads to elevated inhibitory outflow from the basal ganglia and consequent movement disorders. Should this SARS-CoV-2-induced scenario be confirmed to operate in neuronal cells, it would contribute to explaining in part the mechanisms through which the virus causes neurological manifestations that are reminiscent of parkinsonism. Again, this same mechanism might explain the occurrence of olfactory disturbances as a common feature in early parkinsonism and COVID-19, since D2DR was recently documented to induce a tonic inhibition on olfactory abilities within mice olfactory neurons [63]. At the same time, D2DR agonists improve olfactory function and neuroinflammatory injury in olfactory bulb neurons through inhibition of TLR4, IL-1β, and IL-6 [64]. This is key as, similar to the lungs, the olfactory bulb represents both a source of DA pathways and a potential gate for SARS-CoV-2 entry and subsequent propagation in the CNS. Currently, DA receptor agonists are the first choice of treatment for PD patients, which delays the onset of L-DOPA therapy [65]. The DA receptor agonists are also used in a combination with L-DOPA to treat motor complications in advanced stages of PD [65]. This might have important implications in the treatment of PD patients diagnosed with COVID-19, given the antiviral effect of DR agonists documented in the present study.

In summary, although the specific molecular and cellular mechanisms bridging DA activity and regulation of IFN pathways remain to be elucidated, we disclose a previously unappreciated correlation among DA and Type-I IFNs which may be disrupted by SARS-CoV-2 for host cell invasion and replication. In light of the multisystem effects of DA, our findings are expected to pave the way for a novel stream of research focused on the role of the DA system in COVID-19 multi-organ pathology. We firmly believe that this deserves to be investigated in other experimental models and cell types, hopefully contributing to uncovering novel therapeutic targets against SARS-CoV-2.

## Data Availability

Data that support the findings of the present study are available upon reasonable request to the corresponding author.

## Supplementary Materials

The following supporting information can be downloaded at: https://www.mdpi.com/article/10.3390/cells11101691/s1, Table S1: Primer sequences employed in the study; Figure S1: MTT cell viability at 48 h post-infection following treatment with DA at various doses; Figure S2: MTT cell viability at 48 h post-infection following treatment with D1DR or D2DR agonist/antagonist (10 and 50 μM) alone and in combination with 50 μM DA; Figure S3: Trypan Blue cell exclusion assay at 48 h post-infection following treatment with 50 μM DA, or D1DR or D2DR agonist/antagonist 10 μM, either alone and in combination with 50 μM DA. Figure S4: Representative images of combined immunofluorescence for D1DR and SARS-CoV-2 S protein at 48 h post-infection; Figure S5: Immunofluorescence negative controls. Figure S6: MTT cell viability at 48 h post- SARS-CoV-2 infection following treatment with Type-I IFNs at 10 and 100 IU/mL; Figure S7: Representative qPCR melting curves for the expression of the DA-related genes TH, DAT, DDC, D1DR, and D2DR in CaLu-3 cells at 48 h post-infection.

## Abbreviations

CREB	cAMP Response Element-Binding protein
D1DR	D1 Dopamine Receptor
D2DR	D2 Dopamine Receptor
DA	Dopamine
DAT	Dopamine Transporter
DDC	L-Dopa-Decarboxylase
GSK3-β	Glycogen Synthase Kinase 3-beta
HMGB1	High-Mobility Group Box 1
IFN	Interferon
IL-1b	Interleukin 1 beta
IL-6	Interleukin 6
IRF3	Interferon Regulatory Factor 3
MAPK3/ERK1	Mitogen-activated Protein Kinase 3
MOI	Multiplicity of Infection
MTT	3-(4,5-dimethylthiazol-2-yl)-2,5-diphenyltetrazolium bromide
MX-A	Human myxovirus resistance protein 1
NLRP3	NLR family pyrin domain containing 3 Inflammasome
PKA	cAMP-dependent Protein Kinase A
SARS-CoV-2	Severe Acute Respiratory Syndrome-Coronavirus-2
TCID_50_	50% Tissue Culture Infectious Dose
TH	Tyrosine Hydroxylase
TLR4	Toll-like Receptor 4
