# Supplementary material for: Dopamine Reduces SARS-CoV-2 Replication In Vitro through Downregulation of D2 Receptors and Upregulation of Type-I Interferons
Source: Cells. 2022 May 19;11(10):1691. doi: 10.3390/cells11101691 (PMC9139638; doi:10.3390/cells11101691)
Supplement: Supplementary file 1 [file cells-11-01691-s001.zip › cells-1704007-supplementary.pdf]

Supplementary material

Dopamine reduces SARS-CoV-2 replication *in vitro* through downregulation of D2 receptors and upregulation of Type-I Interferons.

Fiona Limanaqi[1,2,*], Silvia Zecchini[2], Borana Dino[2], Sergio Strizzi[2], Gioia Cappelletti[2], Olga Utyro[2], Claudia Vanetti[2], Micaela Garziano[1,2], Irma Saulle[2], Mario Clerici[1,3] and Mara Biasin[2,*]

[1]Department of Pathophysiology and Transplantation, University of Milan, Via Francesco Sforza, Milan, Italy; fiona.limanaqi@unimi.it; micaela.garziano@unimi.it; mario.clerici@unimi.it;
[2]Department of Biomedical and Clinical Sciences L. Sacco, University of Milan, Via G.B. Grassi, Milan, Italy; fiona.limanaqi@unimi.it; silvia.zecchini@unimi.it; borana.dino@studenti.unimi.it; sergio.strizzi@unimi.it; gioia.cappelletti@unimi.it; olga.utyro@unimi.it; claudia.vanetti@unimi.it; irma.saulle@unimi.it; mara.biasin@unimi.it
[3]IRCCS Fondazione Don Carlo Gnocchi, 20148 Milano, Italy. mario.clerici@unimi.it
*Correspondence: Fiona Limanaqi (fiona.limanaqi@unimi.it) and Mara Biasin (mara.biasin@unimi.it)

Table S1. Primer sequences employed in the study.

Gene	FW 5′-3′	RV 5′-3′
GAPDH	CGGATTTGGTCGTATTGGG	GCTTCCCGTTCTCAGCCTTG
D1DR	ATGGACGGGACTGGGCTGGT	GGAGCGTGGACAGGATGAGCA
D2DR	AGCCTCCACTCTCCGCCTG	CCACTAAAGGGAACTGTACTCACC
TH	CGACCCTGACCTGGACTTGGA	GGCAATCTCCTCGGCGGTGT
DAT	AGCCTGCCTGGGTCCTTTCG	AGTGGCGGAGCGTGAACTGG
IFNB	GACGCCGCATTGACCATCTA	GACATTAGCCAGGAGGTTCTCA
MX-A	CCAGAGGCAGGAGACAATCAGC	TCTTCAGGTGGAACACGAGGTT
IRF3	GACAAGGAAGGAGGCGTGTTTG	CAGAGGGCATAGCGTGGTGAG
IL-1β	TTCTGCTTGAGAGGTGCTGATG	TGTCCTGCGTGTTGAAAGATGA
IL4	CAGCAGTTCCACAGGCACAA	TCTGGTTGGCTTCCTTCACA

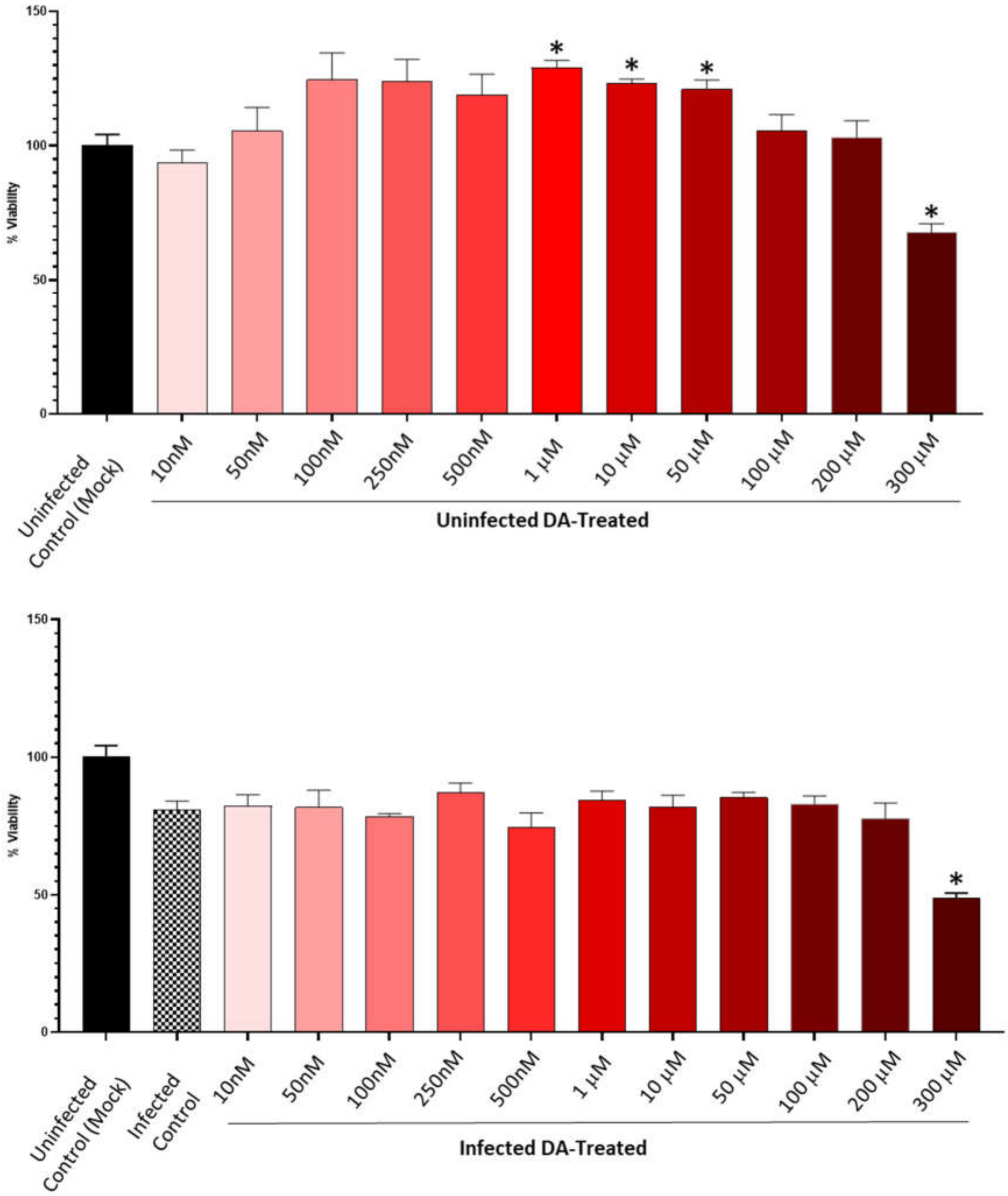

Figure S1. MTT cell viability at 48 h post-infection following treatment with dopamine at various doses. Dopamine (DA) does not induce cytotoxic effects up to 200 µM doses in CaLu-3 cells in either Uninfected (Mock, upper panel) or SARS-CoV-2-infected (lower panel) cells. SARS-CoV-2 per se induced a slight, yet not significant reduction of cell viability compared with Uninfected Control (Mock) cells. At 48 h post- Mock and SARS-CoV-2 infection, 30 µl of MTT (0.5 mg/ml) were added to each well under sterile conditions, and the 96-well plates were incubated for 4 h at 37°C. Supernatants were removed, and dimethyl sulfoxide (100 µl/well) was added. The plates were then agitated on a plate shaker for 5 min. The absorbance of each well was measured at 490 nm with a Bio-Rad automated EIA analyzer (Bio-Rad Laboratories, Hercules, CA, USA). The viability of Uninfected Control cells (Mock) was considered 100%, while the other conditions were expressed as percentages of control. Upper Panel *$p < 0.05$ vs Uninfected Control (Mock); Lower Panel *$p < 0.05$ vs Infected Control.

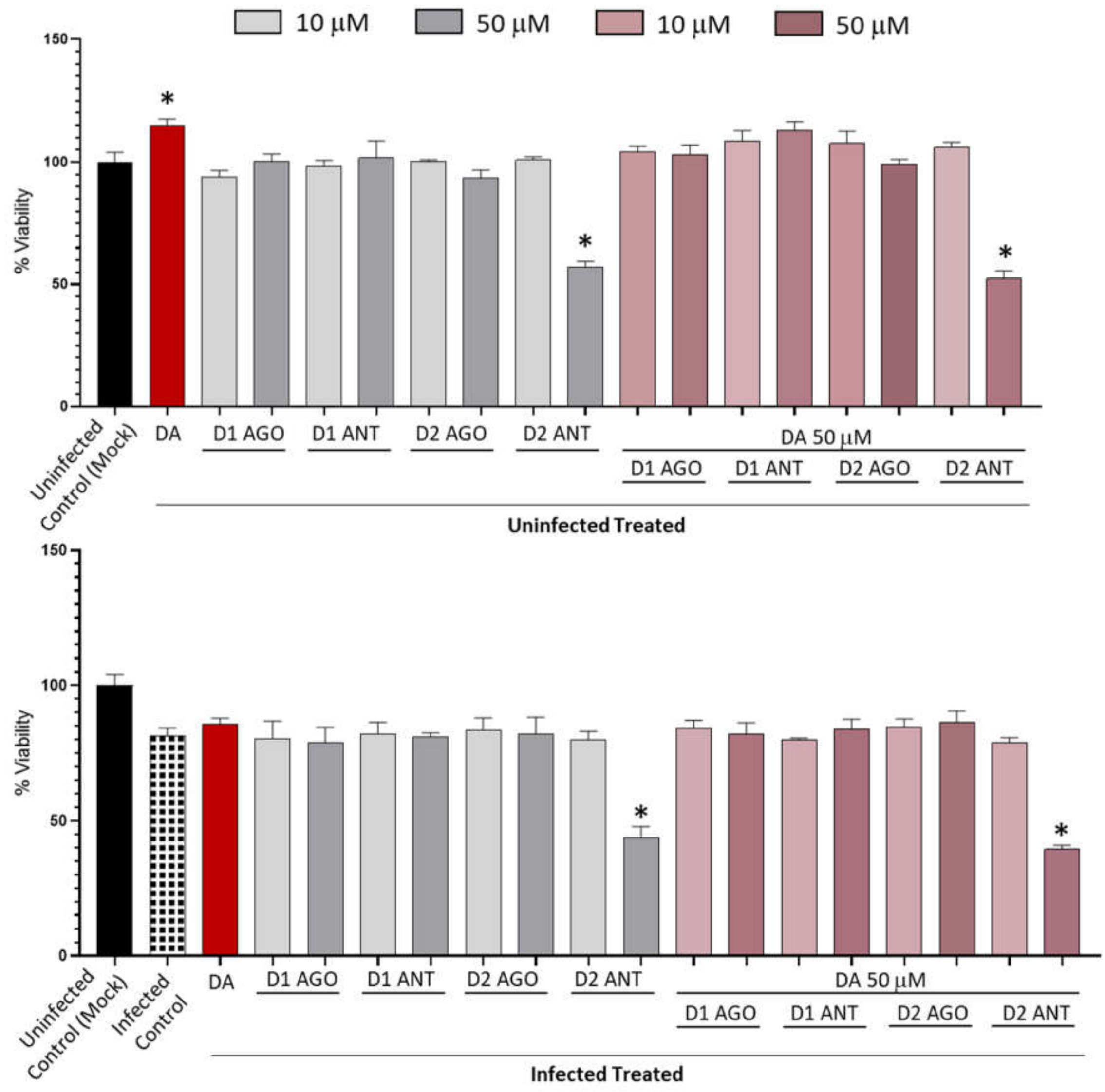

Figure S2. MTT cell viability at 48 h post-infection following treatment with D1DR or D2DR agonists/antagonists alone and in combination with DA. In both Uninfected (Mock, Upper panel) and SARS-CoV-2 infected (Lower panel) cells, administration of D1DR or D2DR agonists and antagonists at 10μM, either alone or in combination with DA 50μM, does not produce any cytotoxic effects, while the D2DR antagonist at the dose of 50μM either alone or in combination with DA produces frank cytotoxicity compared to both Uninfected Control (Upper panel) and SARS-CoV-2-Infected Control (Lower panel). SARS-CoV-2 infection per se produces a slight yet not significant reduction in cell viability compared with Uninfected Control. At 48 h post- Mock and SARS-CoV-2 infection, 30 μl of MTT (0.5 mg/ml) were added to each well under sterile conditions, and the 96-well plates were incubated for 4 h at 37°C. Supernatants were removed, and dimethyl sulfoxide (100 μl/well) was added. The plates were then agitated on a plate shaker for 5 min. The absorbance of each well was measured at 490 nm with a Bio-Rad automated EIA analyzer (Bio-Rad Laboratories, Hercules, CA, USA). The viability of Uninfected Control cells (Mock) was considered 100%, while the other conditions were expressed as percentages of control. Upper panel, *$p < 0.05$ vs Uninfected Control (Mock); Lower panel, *$p<0.05$ vs Infected Control.

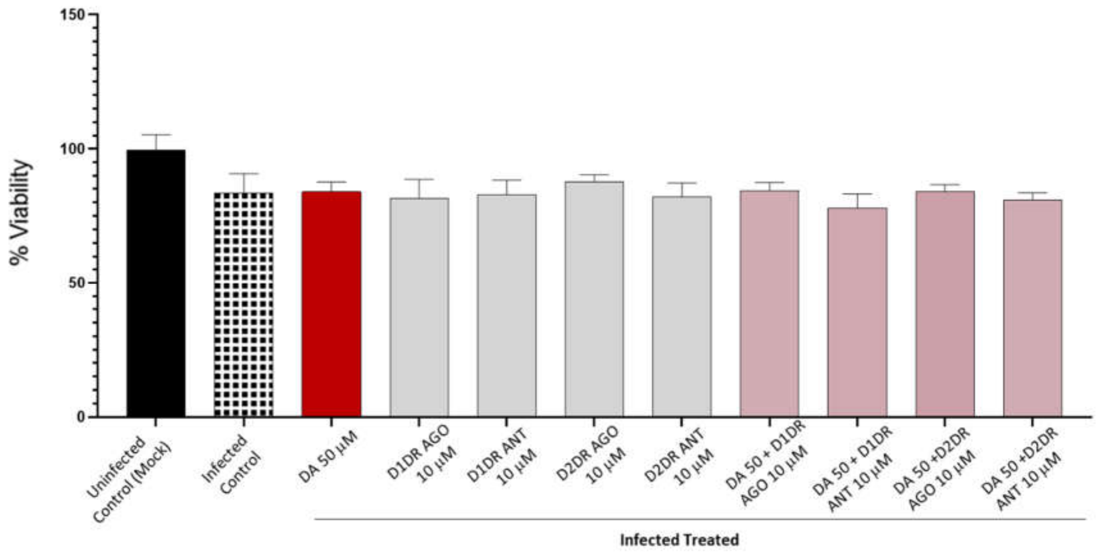

Figure S3. Trypan Blue exclusion assessing cell viability following administration of DA, D1DR or D2DR agonists/antagonists either alone or in combination with DA, at 48 h post SARS-CoV-2 infection in CaLu-3 cells. Treatment with DA 50μM, or D1DR or D2DR agonists and antagonists at 10μM, either alone or in combination with DA 50 μM, does not produce any cytotoxic effects compared with Infected Control (SARS-CoV-2-infected). Compared with Uninfected Control (Mock), SARS-CoV-2 infection per se produces a slight yet not significant reduction in cell viability overlapping with that detected at MTT assay. At 48 h post-infection, cells were dissociated in Cell Dissociation Buffer for 10 min at 37°C. Ten μL of cell suspension were incubated with 10 μL of 0.4% Trypan Blue for 10 min in 96-well plates. Ten μL of the mix were the loaded on chamber slides and counted with the T20 Automated Cell Counter (Bio-Rad Laboratories, Hercules, CA, USA).

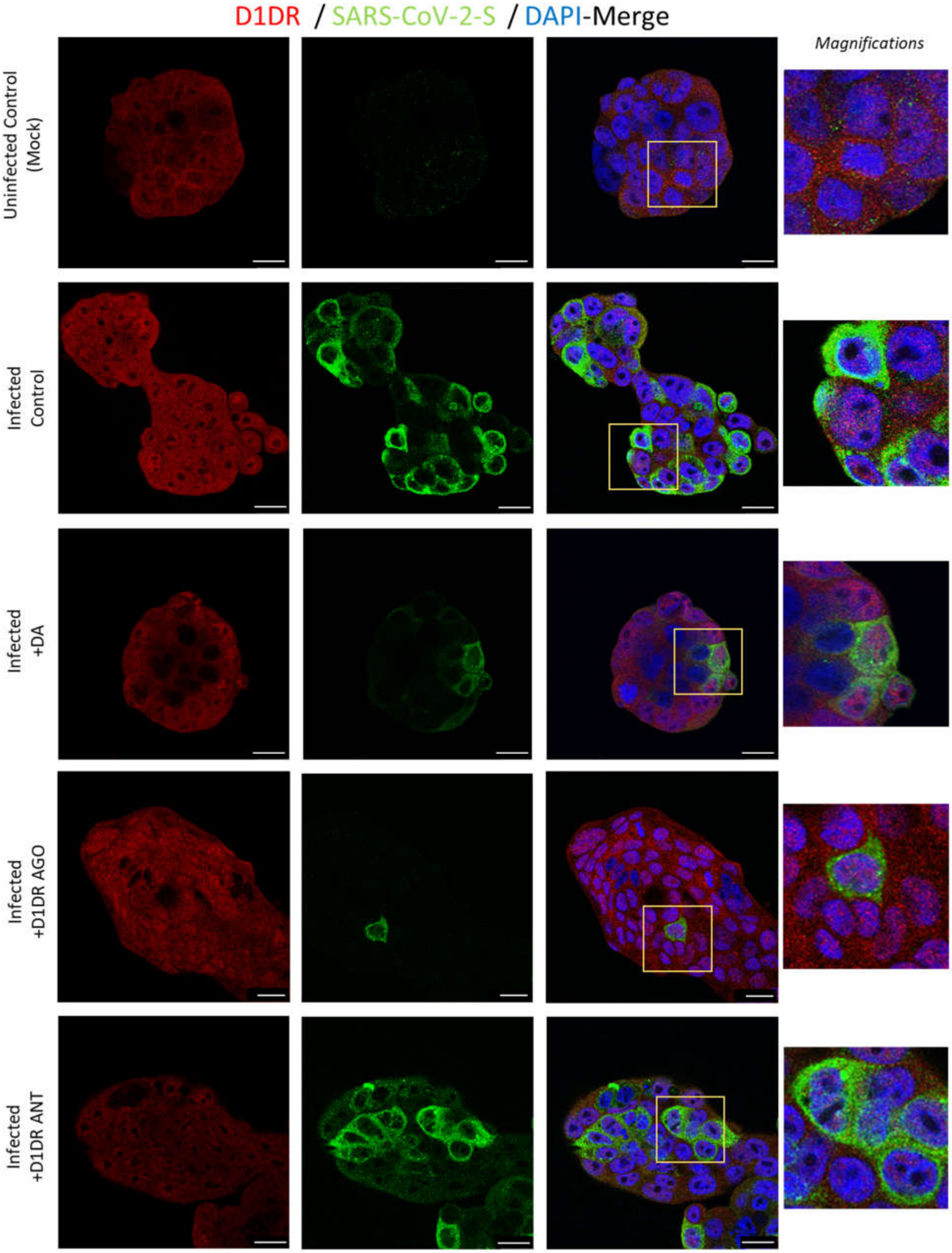

Figure S4. Representative images of combined immunofluorescence for D1DR and SARS-CoV-2 S protein. Although D1DR staining was enhanced by SARS-CoV-2 Infection (Infected Control) compared

with Uninfected Control (Mock), at first glance, no clear-cut correlation emerged between D1DR and SARS-CoV-2 S staining patterns. In SARS-COV-2-infected Control cells, D1DR displayed an extremely diffuse, and scattered, mostly intracellular staining pattern, which did not co-localize with the viral protein. At a closer look, an inverse correlation rather appeared between the localization and intensity of D1DR staining and viral protein. In detail, cells featuring stronger N-immunostaining owned weaker intracellular D1DR staining. Apparently, administration of DA 50 μM, despite reducing SARS-CoV-2 S protein immunostaining, did not produce any evident changes in D1DR staining, apart from slightly inducing such intracellular D1DR clusters. Such an effect was instead magnified following 10 μM D1DR agonist administration, which increased D1DR signal intensity, mostly at intracellular levels, including the cell nucleus. An opposite effect appeared following 10 μM D1DR antagonist, which weakened overall D1DR staining while favoring SARS-CoV-2 replication. At 48 h post-infection, Uninfected cells (Mock) and SARS-CoV-2-Infected cells (both Control and DA/D1DR Agonist/D1DR Antagonist-Treated) were fixed, permeabilized, blocked, and incubated with primary antibodies (Rabbit anti-D1DR bs-1007R, 1:200, Bioss, USA; Mouse anti-S2 Spike SARS-CoV-2, 1:100, GeneTex, Alton Pkwy Irvine, CA, USA), and with secondary antibodies [Goat anti-rabbit Alexa Fluor 647 (ab150079), and Goat anti-mouse Alexa Fluor 488 (ab150113), 1:500, abcam, Cambridge, UK]. Coverslips were mounted on Superfrost glass slides using mounting medium with DAPI (Enzo Life Sciences, Milan, Italy). Confocal imaging was performed with a Leica TCS SP5 AOBS microscope system using a 40X/1.30 oil immersion objective (Leica Microsystems, Wetzlar, Germany). The images shown in figures are representative results from independent experiments. Scale bars correspond to 20 μm.

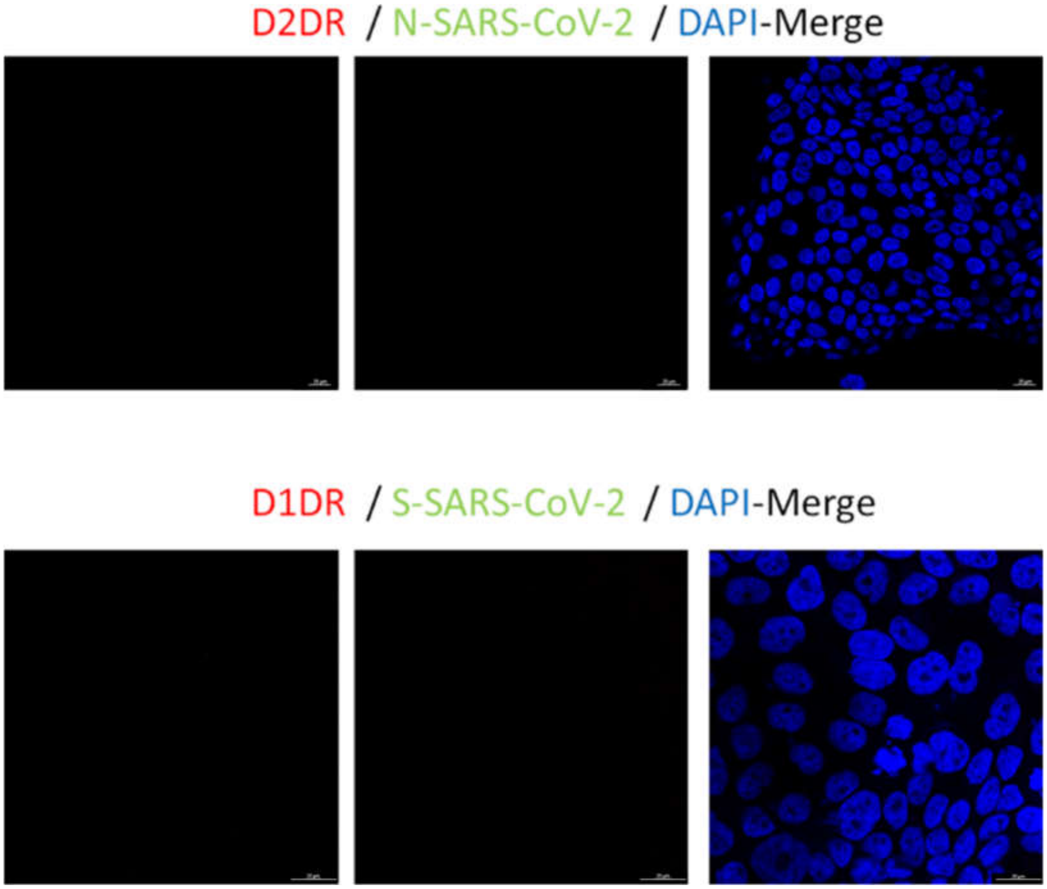

Figure S5. Immunofluorescence negative controls. Negative controls were performed by omitting primary antibodies. Upper panel, CaLu-3 cells were incubated with the secondary antibodies Goat anti-

mouse Alexa Fluor 647 and Goat anti-rabbit Alexa Fluor 488. Lower panel. CaLu-3 cells were incubated with the secondary antibodies Goat anti-rabbit Alexa Fluor 647 and Goat anti-mouse Alexa Fluor 488. At 48 h post-infection, cells were fixed, permeabilized, blocked in 5% BSA-PBS, and incubated for 1 h at RT with 1% BSA-PBS in the absence of primary antibodies. Cells were washed in PBS and incubated for 45 min at RT with secondary antibodies [Goat anti-mouse Alexa Fluor 488 (ab150113) or 647 (ab150115), or Goat anti-rabbit Alexa Fluor 488 (ab150077) or 647 (ab150079), 1:500, abcam, Cambridge, UK]. After washing, coverslips were mounted on Superfrost glass slides using mounting medium with DAPI (Enzo Life Sciences, Milan, Italy). Confocal imaging was performed with a Leica TCS SP5 AOBS microscope system using a 40X/1.30 oil immersion objective (Leica Microsystems, Wetzlar, Germany). The images shown in figures are representative results from independent experiments. Scale bars correspond to 20 μm.

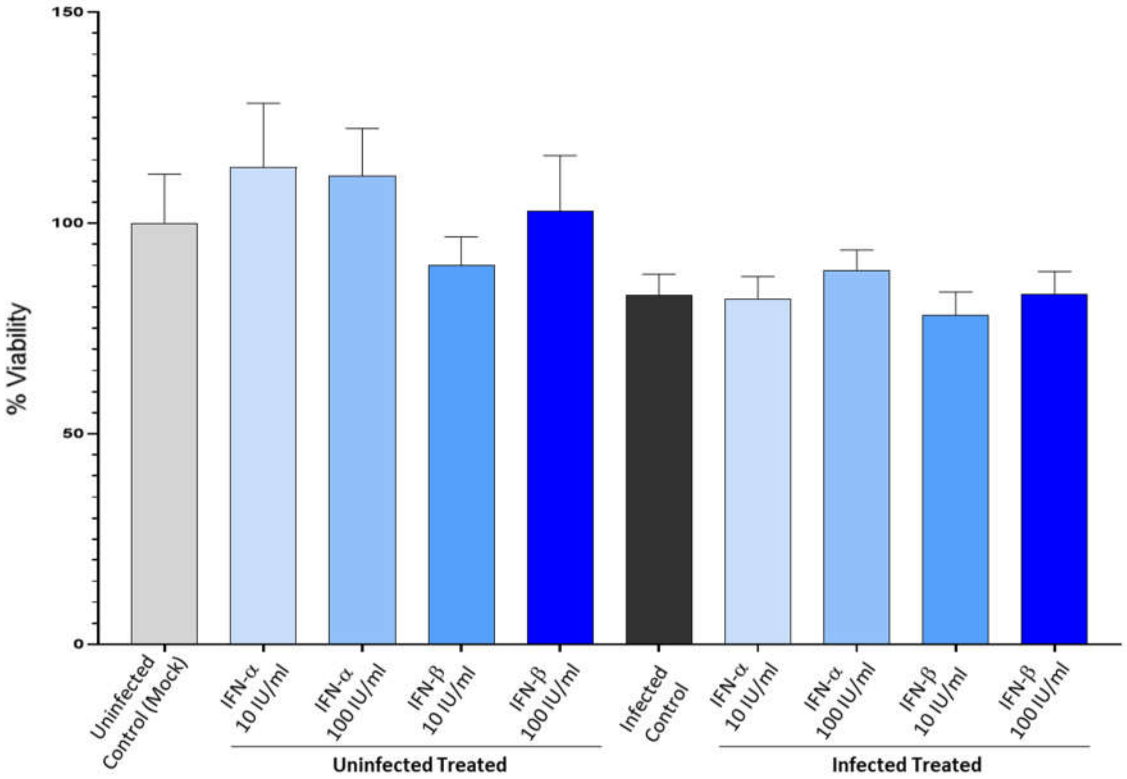

Figure S6. MTT cell viability assessing IFN-induced toxicity at 48 h post-mock and SARS-CoV-2 infection in CaLu-3 cells. In both Uninfected and SARS-CoV-2 infected cells, administration of IFN-α or IFN-β at the doses of 10 and 100 IU/mL does not produce any cytotoxic effects compared to Uninfected Control and SARS-CoV-2-Infected Control, respectively. SARS-CoV-2 infection per se produces a slight yet not significant reduction in cell viability compared with Uninfected Control. At 48 h post- Mock- and SARS-CoV-2 infection, 30 μl of MTT (0.5 mg/ml) were added to each well under sterile conditions, and the 96-well plates were incubated for 4 h at 37°C. Supernatants were removed, and dimethyl sulfoxide (100 μl/well) was added. The plates were then agitated on a plate shaker for 5 min. The absorbance of each well was measured at 490 nm with a Bio-Rad automated EIA analyzer (Bio-Rad Laboratories, Hercules, CA, USA). The viability of Uninfected Control cells (Mock) was considered 100%, while the other conditions were expressed as percentages of control.

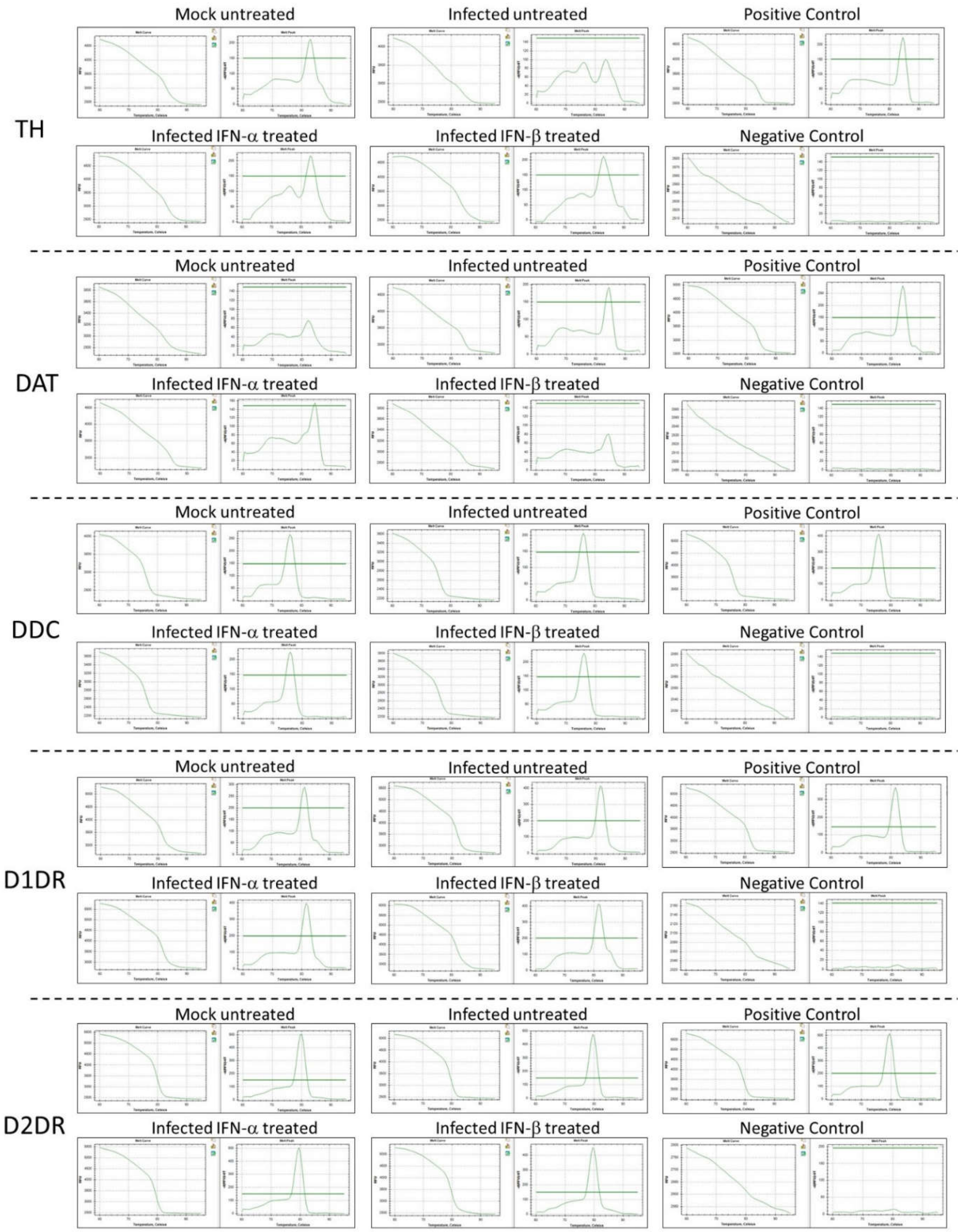

Figure S7. Representative qPCR melting curves for the expression of the DA-related genes TH, DAT, DDC, D1DR, and D2DR in CaLu-3 cells at 48 h post-infection**.** Melting curves are representative from Uninfected Control (Mock untreated), SARS-CoV-2 Infected Control (Infected untreated), and SARS-CoV-2-Infected IFN-treated cells. Negative controls (distilled water, and total RNA sample RT-negative), as well as Positive controls (cDNA from human DA-iPSC cells) were included in each run. The images are representative of multiple real time qPCRs from different experiments.
